# Identification of genetic variants of the industrial yeast *Komagataella phaffii (Pichia pastoris)* that contribute to increased yields of secreted heterologous proteins

**DOI:** 10.1371/journal.pbio.3001877

**Published:** 2022-12-15

**Authors:** Benjamin Offei, Stephanie Braun-Galleani, Anjan Venkatesh, William T. Casey, Kevin E. O’Connor, Kevin P. Byrne, Kenneth H. Wolfe

**Affiliations:** 1 UCD Conway Institute and School of Medicine, University College Dublin, Dublin, Ireland; 2 School of Biochemical Engineering, Pontificia Universidad Católica de Valparaíso, Valparaíso, Chile; 3 Bioplastech Ltd., NovaUCD, Belfield Innovation Park, University College Dublin, Dublin, Ireland; 4 UCD Earth Institute and School of Biomolecular & Biomedical Science, University College Dublin, Dublin, Ireland; 5 BiOrbic Bioeconomy SFI Research Centre, University College Dublin, Dublin, Ireland; Stanford University, UNITED STATES

## Abstract

The yeast *Komagataella phaffii* (formerly called *Pichia pastoris*) is used widely as a host for secretion of heterologous proteins, but only a few isolates of this species exist and all the commonly used expression systems are derived from a single genetic background, CBS7435 (NRRL Y-11430). We hypothesized that other genetic backgrounds could harbor variants that affect yields of secreted proteins. We crossed CBS7435 with 2 other *K*. *phaffii* isolates and mapped quantitative trait loci (QTLs) for secretion of a heterologous protein, β-glucosidase, by sequencing individual segregant genomes. A major QTL mapped to a frameshift mutation in the mannosyltransferase gene *HOC1*, which gives CBS7435 a weaker cell wall and higher protein secretion than the other isolates. Inactivation of *HOC1* in the other isolates doubled β-glucosidase secretion. A second QTL mapped to an amino acid substitution in *IRA1* that tripled β-glucosidase secretion in 1-week batch cultures but reduced cell viability, and its effects are specific to this heterologous protein. Our results demonstrate that QTL analysis is a powerful method for dissecting the basis of biotechnological traits in nonconventional yeasts, and a route to improving their industrial performance.

## Abstract 2

The yeast Komagataella phaffii (Pichia pastoris) is widely used as a host for secretion of heterologous proteins in biotech applications, but all the commonly used expression systems are derived from a single genetic background. Here, genetic mapping in K. phaffii identifies gene variants that affect the yield of secreted foreign proteins.

## Introduction

Because they are microbial eukaryotes, yeasts offer many beneficial attributes for heterologous protein production that are absent from cellular platforms based on bacterial or mammalian cells. Although *Saccharomyces cerevisiae* remains a popular expression host, “nonconventional” yeast species have eclipsed it in recent decades. These species offer advantages over *S*. *cerevisiae* such as growth to very high cell densities, thermotolerance, and fewer endogenous secreted proteins. Foremost among the nonconventional yeasts is *Komagataella phaffii*, which has become very widely used for secretion of heterologous proteins [1–4]. *K*. *phaffii* is one of the 2 yeast species that were formerly called *Pichia pastoris* until they were recognized as separate species in 2009 [5]. *K*. *phaffii* is used to produce marketed therapeutic proteins including human insulin [6], interferon alpha [7], kallikrein inhibitor [8], and plasmin [8], as well as vaccines [6,9] and antibodies [10,11]. Additionally, it is used to produce enzymes for the food and feed industries [12]. All these proteins are secreted.

Many approaches have been taken to try to further improve the industrial performance of *K*. *phaffii* as a host for heterologous protein expression, including bioprocess engineering, expression cassette engineering, and host cell engineering [3,13]. Previous efforts to modify the host cell’s genome have targeted particular pathways, such as the development of protease-free strains [14], strains that overexpress unfolded protein response pathway genes [15], and *och1* strains deficient in hypermannosylation of secreted proteins [16–18]. In contrast to these targeted approaches, quantitative trait locus (QTL) analysis has the advantage that it does not require any prior hypotheses regarding which genes will contribute to the phenotype of interest. Most previous yeast QTL analyses have been conducted in *S*. *cerevisiae* [19], and they led to the identification of genes responsible for several industrially relevant polygenic phenotypes in that species [20–24]. QTL mapping has been applied to only a few other yeast species, including *Schizosaccharomyces* [25], *Lachancea* [26,27], and *Cryptococcus* [28]. *K*. *phaffii* seems particularly suitable for QTL analysis because all the strains currently used for heterologous protein production are derived from the same progenitor strain (called CBS7435 or NRRL Y-11430) [5,29–31], so it seems likely that other genetic backgrounds may contain beneficial alleles that could be transferred into production strains by genome editing. Nevertheless, QTL approaches have remained unexplored in *K*. *phaffii* and other nonconventional yeast species used in biotechnology.

Here, we mapped QTLs affecting secretion of a heterologous protein in *K*. *phaffii* and identified the causative nucleotides underlying 2 QTLs. To achieve high resolution, we combined 2 techniques that have previously only been used separately in *S*. *cerevisiae* QTL studies: screening a large number of meiotic segregants to select ones with extreme phenotypes before genotyping [20,32], and sequencing the genome of each selected segregant individually instead of sequencing bulk DNA from each phenotypic pool [33,34].

## Results

### Construction of parental BGL-secreting strains

**Fig 1:**
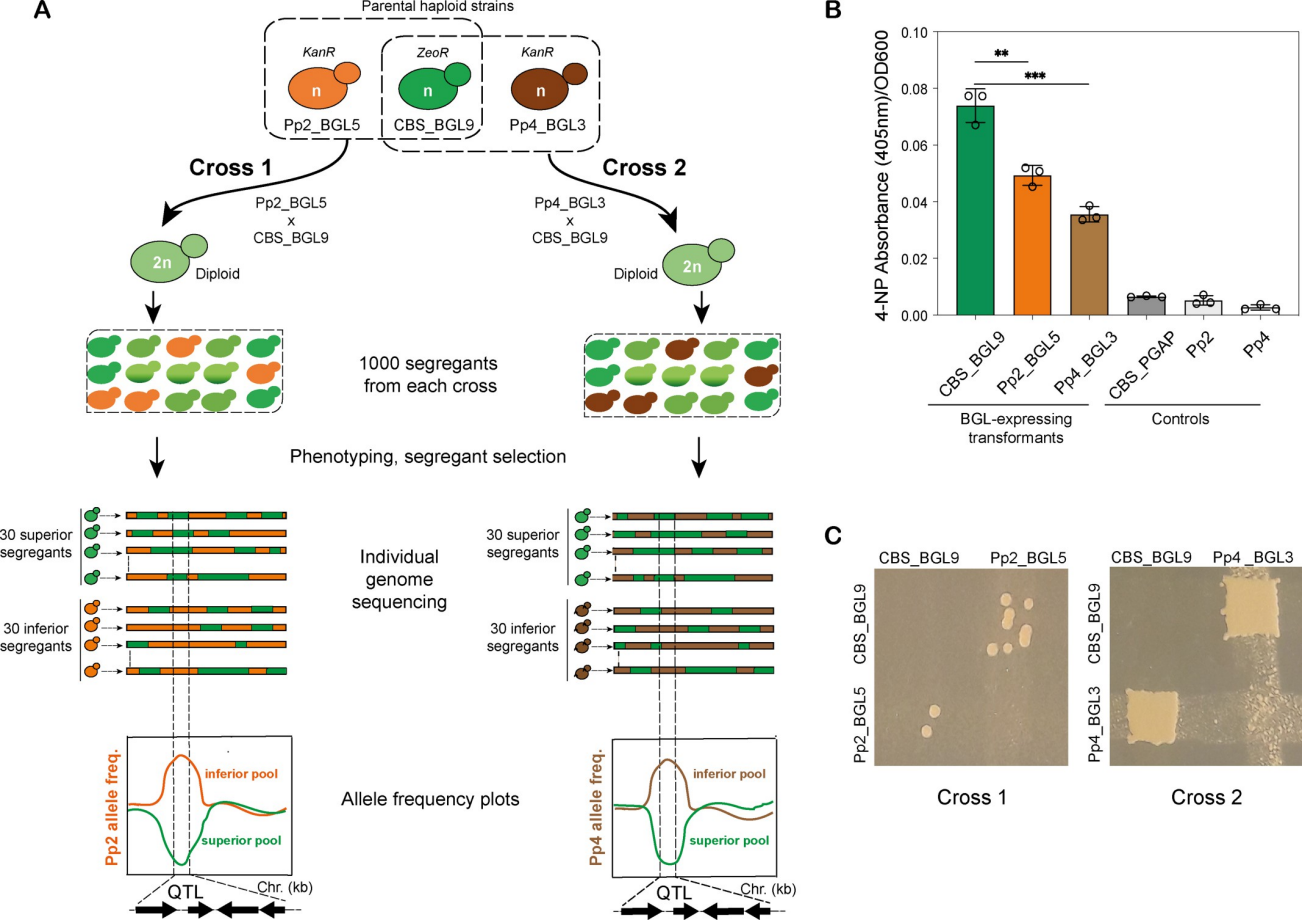
Parental strains and design of genetic crosses. **(A)** Experimental design. Two crosses were made between a derivative of CBS7435 (CBS_BGL9) and derivatives of Pp2 (Pp2_BGL5) or Pp4 (Pp4_BGL3). These parental strains each contained a BGL expression cassette and *KanR* or *ZeoR* marker, integrated at the *GAPDH* locus. The resulting diploid interstrain hybrids were sporulated and 1,000 haploid meiotic segregants were isolated from each cross by random spore isolation. BGL secretion in each segregant was assayed and used to choose pools of 30 superior segregants and 30 inferior segregants from each cross. Genomes of the segregants in each pool were then sequenced individually, after which SNP allele frequency plots were constructed for each pool, enabling identification of genomic regions (QTLs) segregating preferentially with contrasting BGL secretion phenotypes in each cross. (**B**) Quantitative assessment of BGL secretion in the parental strains and controls (CBS_PGAP is a derivative of CBS7435 containing an integrated empty pGAPZα vector; Pp2 and Pp4 are wild-type strains). Bars represent mean data from 3 independent cultures of each strain (open circles). Error bars show standard deviation. Significant differences in BGL secretion between CBS_BGL9 and the other parental strains were tested with unpaired *t* test and are indicated by asterisks (**, *P* = 0.0035; and ***, *P* = 0.0005). Numerical data are listed in S1 Data. **(C**) Formation of diploids by crossing. Diploid colonies formed by mating grow at the intersections between streaks of the haploid parental strains, when replica plated onto selective media containing zeocin plus geneticin. BGL, β-glucosidase; QTL, quantitative trait locus; SNP, single-nucleotide polymorphism.

Only 4 different natural isolates of *K*. *phaffii* are known, but they harbor substantial genetic and phenotypic diversity [30,31]. Isolates Pp2 (NRRL Y-17741) and Pp4 (NRRL YB-378) both differ from the reference strain CBS7435 by about 42,000 single-nucleotide polymorphisms (SNPs) and are similarly divergent from each other [30,31]. To maximize our ability to find variants of interest, we designed a mating scheme with 2 crosses between these different genetic backgrounds: Pp2 × CBS7435 (Cross 1), and Pp4 × CBS7435 (Cross 2) (Fig 1A).

We chose the β**-**glucosidase (BGL) enzyme of the filamentous fungus *Thermoascus aurantiacus* as a model secreted protein, because it can be assayed readily in microtiter plates and has been expressed previously in *K*. *phaffii* [35]. We cloned the *T*. *aurantiacus BGL* gene into a pGAPZα plasmid (Invitrogen), creating a BGL expression cassette with a zeocin resistance gene (*ZeoR*) for integration at the constitutive P_*GAP*_ (*GAPDH*) promoter locus of CBS7435. Additionally, a derivative of this cassette harboring a geneticin resistance gene (*KanR*) instead of *ZeoR* was integrated at the P_*GAP*_ loci of Pp2 and Pp4 (S1 Fig). Transformants were assessed for extracellular BGL secretion using both qualitative 4-MUG assays (UV fluorescence due to hydrolysis of 4-methylumbelliferyl-β-D-glucuronide to 4-methylumbelliferone) and quantitative 4-NPG assays (optical absorbance at 405 nm due to hydrolysis of 4-nitrophenyl-β-D-glucopyranoside to 4-nitrophenol (4-NP)). BGL activity was detected in supernatants of most transformants carrying the BGL expression construct and absent in controls (S1 Fig).

We then selected 1 BGL*-*expressing transformant of each of CBS7435 (CBS_BGL9), Pp2 (Pp2_BGL5), and Pp4 (Pp4_BGL3) for use as parents in genetic crosses. Additionally, we compared BGL secretion per unit biomass in these 3 transformants after a 96-hour cultivation period in 400 μl cultures. The CBS7435-derived strain CBS_BGL9 was superior in terms of BGL secretion and produced approximately twice as much 4-NP per cell as Pp2_BGL5 and Pp4_BGL3 (Fig 1B).

### Genetic crosses and screening of meiotic segregants

In each genetic cross (Fig 1A), both parents express BGL from the constitutive P_*GAP*_ promoter at the *GAPDH* locus, but their expression constructs are tagged with different markers, enabling us to select for double antibiotic resistance (*ZeoR KanR*) in diploids formed by mating. Mating efficiency was much lower in Cross 1 than in Cross 2 (Fig 1C), but we obtained diploids from both crosses and confirmed their ploidy by flow cytometry (S2 Fig). Sporulation followed by random spore isolation [36] yielded 2 main types of haploid spore clones, *KanR* and *ZeoR*, resulting from the allelic location of the antibiotic resistance markers. A small number of spore clones that exhibited resistance to both antibiotics were discarded as they likely represented clumps of spores [36]. We isolated 1,000 meiotic segregants (500 *KanR* and 500 *ZeoR*) from each cross.

**Fig 2:**
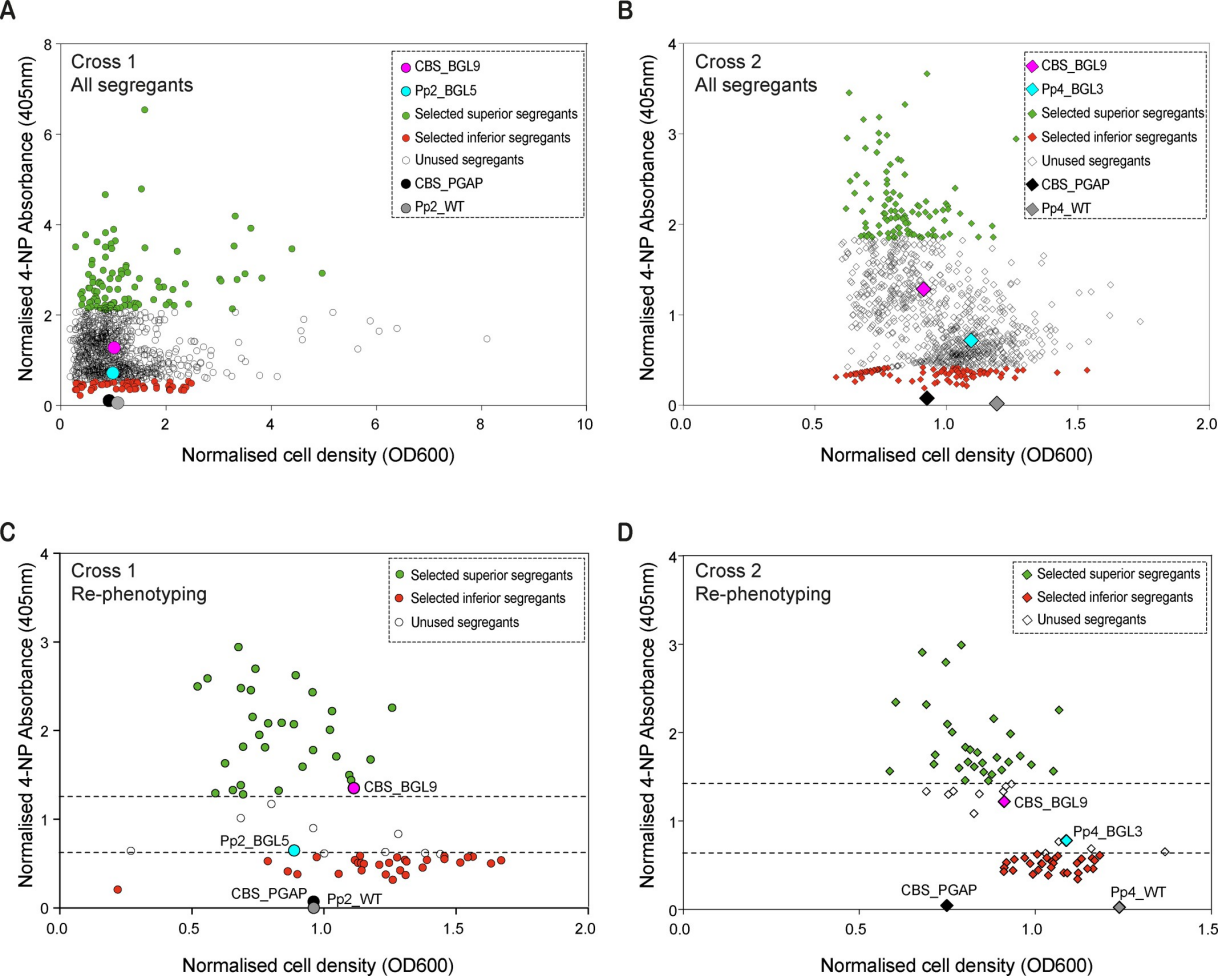
Phenotypic analysis of meiotic segregants. **(A, B)** Dotplots showing normalized BGL secretion and cell density of all 1,000 segregants obtained from (**A)** Cross 1 (Pp2_BGL5 × CBS_BGL9) and (**B**) Cross 2 (Pp4_BGL3 × CBS_BGL9). Data are shown for the 2 parental strains in each cross, as well as for strains lacking the BGL expression construct (CBS_PGAP and wild-type Pp2 or Pp4). Green and red symbols indicate the initial sets of superior (green) and inferior (red) segregants selected from each cross for rephenotyping and ploidy checking. (**C, D)** Rephenotyping results for high and low BGL-secreting segregants selected from the green and red portions, respectively, in (**A)** and **(B)** and confirmed as haploid by flow cytometry. The final 30 superior and 30 inferior segregants are shown in green and red, respectively, on each plot. Dashed lines indicate cutoff values for selection. Also shown are data from the parental strains and controls (symbols as in panels **A** and **B**). Numerical data are listed in S1 Data.

For initial phenotyping, we grew 4-day 400 μl cultures of all 2,000 segregants and used 4-NP absorbance at 405 nm as a quantitative readout of extracellular BGL secretion. Since we screened these segregants in batches using deep 96-well plates, we corrected for interplate variation by dividing the 4-NP absorbance and cell density of each segregant culture in a batch by the average absorbance of the 2 parental strains, which were cultured alongside each batch of segregants on a plate. Plots of these normalized values show that the segregants exhibit a continuum of BGL secretion, as well as extensive variation in cell density (Fig 2A and 2B). The distribution of BGL secretion levels in the segregants was similar in the 2 crosses and approximated a normal frequency distribution (S3 Fig), suggesting that BGL secretion is a polygenic trait.

To select segregants to be used as QTL mapping populations, we initially chose approximately 35 segregants from the top (superior BGL secretors) and bottom (inferior BGL secretors) of the plots for each cross (Fig 2A and 2B). We rephenotyped these segregants using 4-day 1 ml cultures and used flow cytometry to check that they are haploid (S2 Fig). From this step, we retained 30 superior and 30 inferior segregants from each cross, i.e., the top 3% and bottom 3%, which were haploid and showed consistently high or low BGL secretion (Figs 2C and 2D and S3). For both crosses, almost all 30 segregants in the superior pool showed higher BGL secretion than the superior parent (CBS_BGL9), and all 30 segregants in the inferior pool showed lower BGL secretion than the inferior parent (Pp2_BGL5 or Pp4_BGL3) (Fig 2C and 2D), indicating transgressive segregation of the phenotype. We thus obtained 4 pools (superior and inferior from Cross 1; superior and inferior from Cross 2), with 30 segregants in each. There is a significant negative correlation between BGL secretion and cell density in both crosses, which suggests that there may be a trade-off between secretion and growth rate (Fig 2A and 2B; Pearson’s *r* = −0.48, *p* = <0.0001 for Cross 1 and *r* = −0.68, *p* = <0.0001 for Cross 2). This negative correlation was also seen in the rephenotyping data, although cell density values for individual segregants had low reproducibility between the 2 phenotyping steps (S3 Fig).

### QTL mapping by sequencing individual segregants

**Fig 3:**
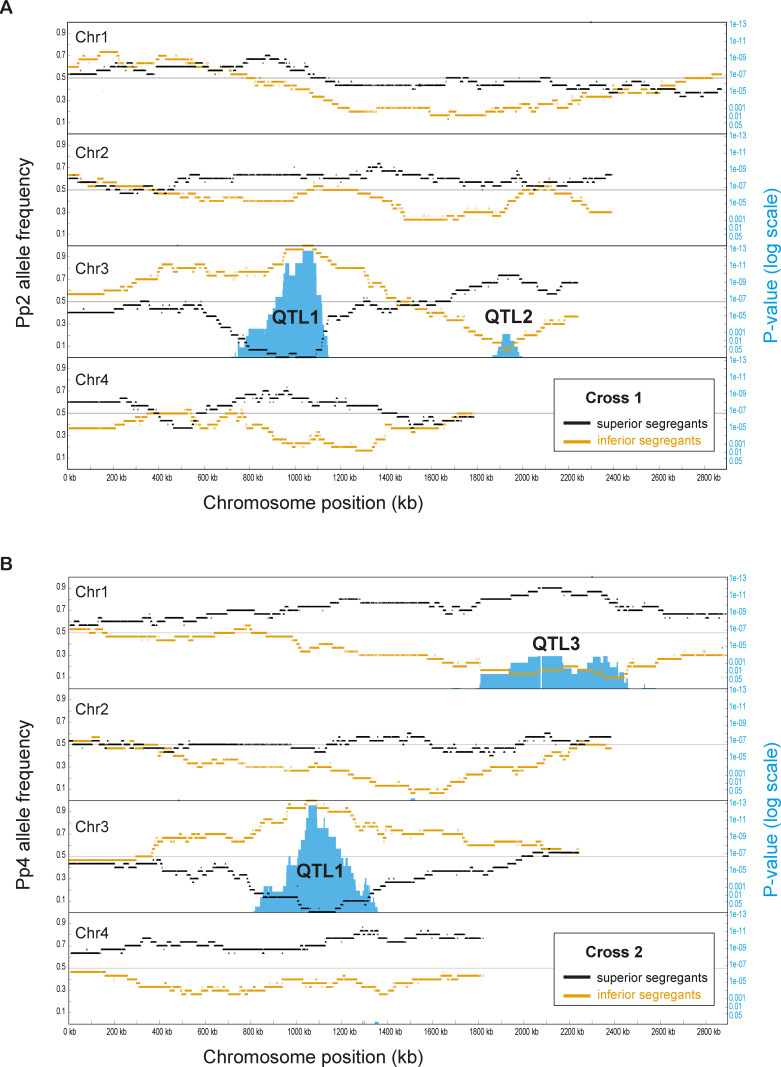
QTL analysis using SNP allele frequencies calculated from genome sequences of individual segregants. **(A**) Cross 1 (Pp2_BGL5 × CBS_BGL9). Left axis: Black dots show the frequency of the Pp2 allele among the 30 superior segregants, and orange dots show its frequency among the 30 inferior segregants, at each of the 42,262 polymorphic sites segregating on the 4 *K*. *phaffii* chromosomes in this cross. The gray horizontal lines at allele frequency 0.5 represent unbiased segregation. Right axis: Blue bars show the statistical significance of bias in allele frequency (log10 scale; Fisher’s exact test with Bonferroni correction for multiple testing over all SNPs on a chromosome). (**B**) Cross 2 (Pp4_BGL3 × CBS_BGL9). Black dots show the frequency of the Pp4 allele among the 30 superior segregants, and orange dots show its frequency among the 30 inferior segregants, at each of the 41,552 polymorphic sites segregating in this cross. Other details are as in (**A)**. Numerical data are listed in S2 Data.

We sequenced the genomes of each of the 120 selected segregants individually, which enables us to calculate the exact allele frequencies of all variants in each pool. Each segregant’s genome was sequenced to approximately 100× coverage (Illumina). After quality filtering, reads were mapped to the CBS7435 reference genome [29] and the genotype of the segregant at each of the approximately 42,000 SNP sites segregating in the cross was determined. For each SNP site, we then calculated the allele frequency of the nonreference allele (i.e., the Pp2 allele in Cross 1, and the Pp4 allele in Cross 2) among the 30 genomes in the superior pool and among the 30 genomes in the inferior pool. These frequencies are plotted versus genomic position, for each pool in each cross (Fig 3A and 3B). Neutral SNP sites are expected to show a nonreference allele frequency of approximately 0.5 in both pools, whereas SNP sites genetically linked to loci affecting BGL secretion should deviate from a 0.5 frequency in opposite directions in the 2 pools.

For comparison, we also used the standard bulk segregant analysis approach of combining equal quantities of biomass from the 30 segregants in each pool, sequencing bulk DNA from this mixture, and using SNP frequencies in the sequencing reads as a proxy for their frequencies in the 30 segregants. The results from this approach (S4 Fig) show the same pattern of variation in allele frequencies along chromosomes as we observe in Fig 3, but with much more noise and consequently lower resolution than we obtained by sequencing segregants individually.

### QTL1 maps to a frameshifted allele of *HOC1* in CBS7435

In both Cross 1 and Cross 2, a prominent QTL (designated QTL1) is apparent near the center of chromosome 3 (Fig 3). In the 43-kb region at the peak of QTL1 in Cross 1, all 30 superior segregants contain the CBS7435 haplotype and all 30 inferior segregants contain the Pp2 haplotype. A statistical test for difference in allele frequencies between the 2 pools reaches a significance of *P* = 1.84 × 10^−13^ at each SNP site in this peak region of QTL1 in Cross 1 (blue bars in Fig 3A). Similarly, at QTL1 in Cross 2, all 30 superior segregants contain the CBS7435 haplotype and all 30 inferior segregants contain the Pp4 haplotype in a 33-kb region at its peak (Fig 3B; *P* = 1.81 × 10^−13^). Thus, in this region of the genome, the CBS7435 allele is superior to both the Pp2 and Pp4 alleles.

**Fig 4:**
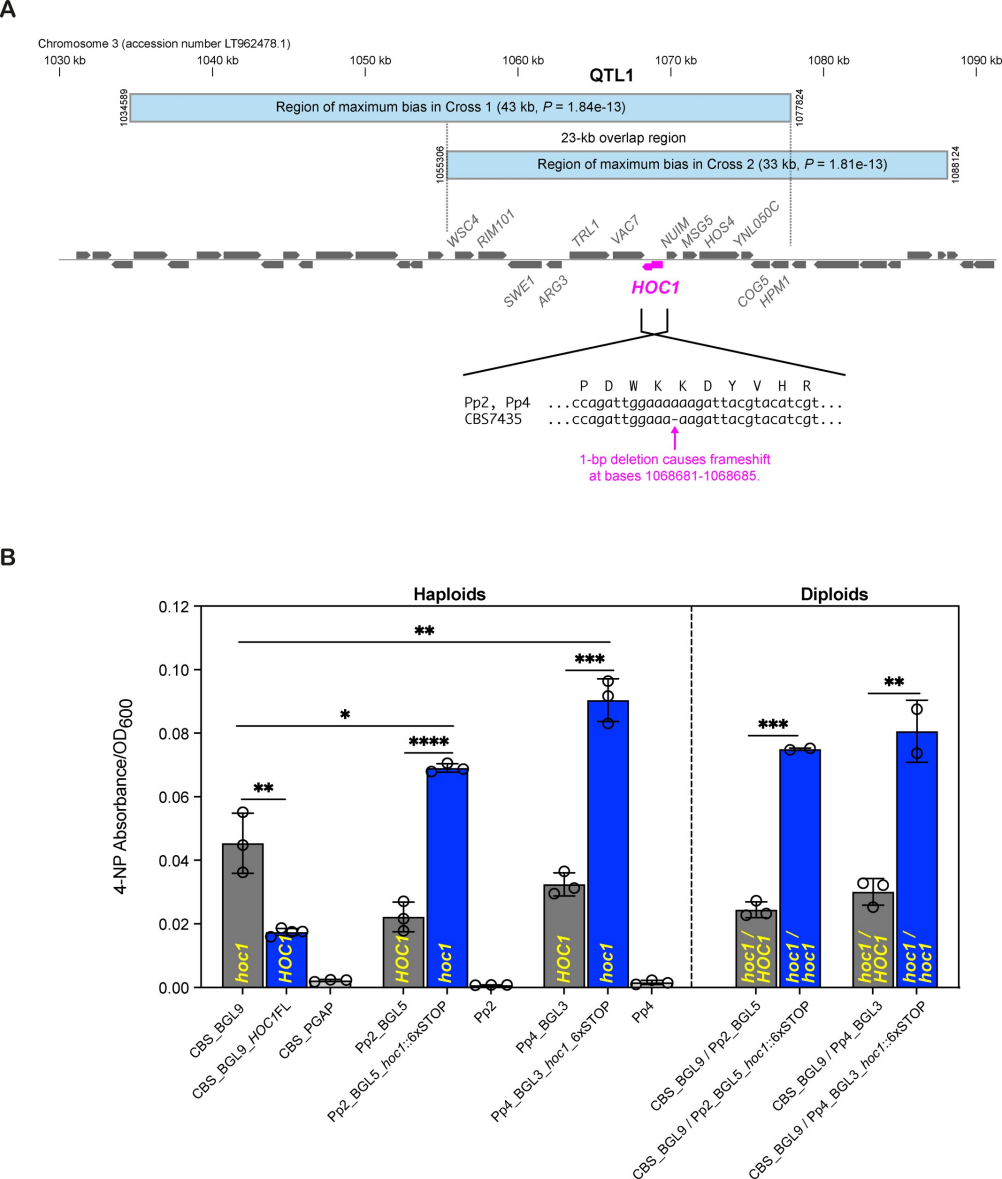
QTL1 maps to a frameshift mutation in *HOC1*. **(A)** Gene map at the peak region of QTL1. Blue bars indicate the regions of maximum segregation bias in each cross. In these regions, the CBS7435 SNP alleles are present in all 30 superior segregants and absent in all 30 inferior segregants. Genes are named according to their *S*. *cerevisiae* orthologs where possible. The inset shows the frameshift mutation in the CBS7435 allele of *HOC1* (systematic gene name BQ9382_C3-3105). (**B**) BGL secretion in strains with *HOC1* edits (blue bars) and their unedited progenitors (gray bars). The relevant genotype of each strain is indicated in yellow. CBS_BGL9_*HOC1*FL is a derivative of CBS_BGL9 in which the *HOC1* gene was restored to functionality by CRISPR/Cas9 editing to remove the frameshift, making it full length; 4 independent cultures of the same edited strain were assayed. For the other 4 edited strains, the Pp2 or Pp4 *HOC1* gene was disrupted by introducing a sequence containing 6 consecutive stop codons (6xSTOP tag) after amino acid position Gly157 in the Hoc1 protein, and 2–3 independently edited clones were assayed. For each of the edited diploids, we recovered and assayed 1 clone in which the Pp2/Pp4 allele was disrupted by a 6xSTOP tag and the CBS7435 allele was disrupted by its original frameshift, and 1 clone in which 6xSTOP tags were inserted into both the Pp2/Pp4 allele and the frameshifted CBS7435 allele. Assays were conducted on 4-day 400 μl cultures. Error bars indicate standard deviation. Significant differences in BGL secretion were tested with unpaired *t* tests (two-tailed) and are indicated by asterisks (*, *P* < 0.05. **, *P* < 0.01. ***, *P* < 0.001 and ****, *P* < 0.0001). Numerical data are listed in S1 Data.

The regions of peak statistical significance in the 2 crosses overlap in a 23-kb interval that contains 13 genes including *HOC1* (Fig 4A). The Pp2 and Pp4 alleles of *HOC1* are intact and code for a 397-residue protein that is well conserved among budding yeasts, but the CBS7435 allele contains a frameshift mutation that truncates the Hoc1 protein to 273 residues and presumably inactivates it. *S*. *cerevisiae HOC1* (“homolog of *OCH1*”) codes for a protein with a mannosyltransferase domain that is a subunit of the Mannan Polymerase II complex, which is located in the Golgi apparatus and extends the α1,6-linked backbone of mannan chains of *N*-glycosylated proteins [37]. Disruption of *S*. *cerevisiae HOC1* causes cell wall defects, so Hoc1 is postulated to function in glycosylation of cell wall proteins [38]. A deletion of *HOC1* was recovered in a genomewide screen for increased protein secretion in *S*. *cerevisiae* [39], and disruption or deletion of another Mannan Polymerase II subunit Mnn10 also increases yields of secreted proteins in *S*. *cerevisiae* and *Kluyveromyces lactis* [40,41], so *K*. *phaffii HOC1* was a strong candidate gene at QTL1.

We verified that the frameshifted *K*. *phaffii hoc1* allele in CBS7435 is the cause of QTL1. Inactivation of the intact Pp2 or Pp4 *HOC1* gene by CRISPR/Cas9 editing [42] more than doubled BGL secretion in Pp2_BGL5 and Pp4_BGL3 haploids, as well as in CBS_BGL9/Pp2_BGL5 and CBS_BGL9/Pp4_BGL3 diploids (Fig 4B). Conversely, correction of the frameshift to repair *HOC1* in haploid CBS_BGL9 halved its BGL secretion. Interestingly, among haploids, BGL secretion was higher in the *hoc1* derivatives of Pp2 and Pp4 than in the CBS7435 background (Fig 4B), suggesting that they have potential as host strains. Using the edited haploid strains, we also found that *K*. *phaffii hoc1* mutants are sensitive to the cell wall-perturbing agent Calcofluor White, whereas *HOC1* strains are resistant (S5 Fig).

### QTL2 maps to *IRA1*

Each cross contains one other QTL that reaches statistical significance: QTL2 on chromosome 3 in Cross 1 (*P* = 1.57 × 10^−3^), and QTL3 on chromosome 1 in Cross 2 (*P* = 1.32 × 10^−4^) (Fig 3). At these QTLs, the superior allele comes from the nonreference parent Pp2 (at QTL2) or Pp4 (at QTL3), so these are alleles with potential to improve protein secretion if introduced into the widely used strain CBS7435. Each of these QTLs is absent in the other cross, which is not surprising because Pp2 and Pp4 are quite divergent from each other [30]. We chose QTL2 for further analysis because it is narrower than QTL3. It is striking that QTL1 and QTL2 are in opposite phases on the same chromosome, so that most of the segregants in both pools in Cross 1 have a crossover in the interval between them (Fig 3A).

**Fig 5:**
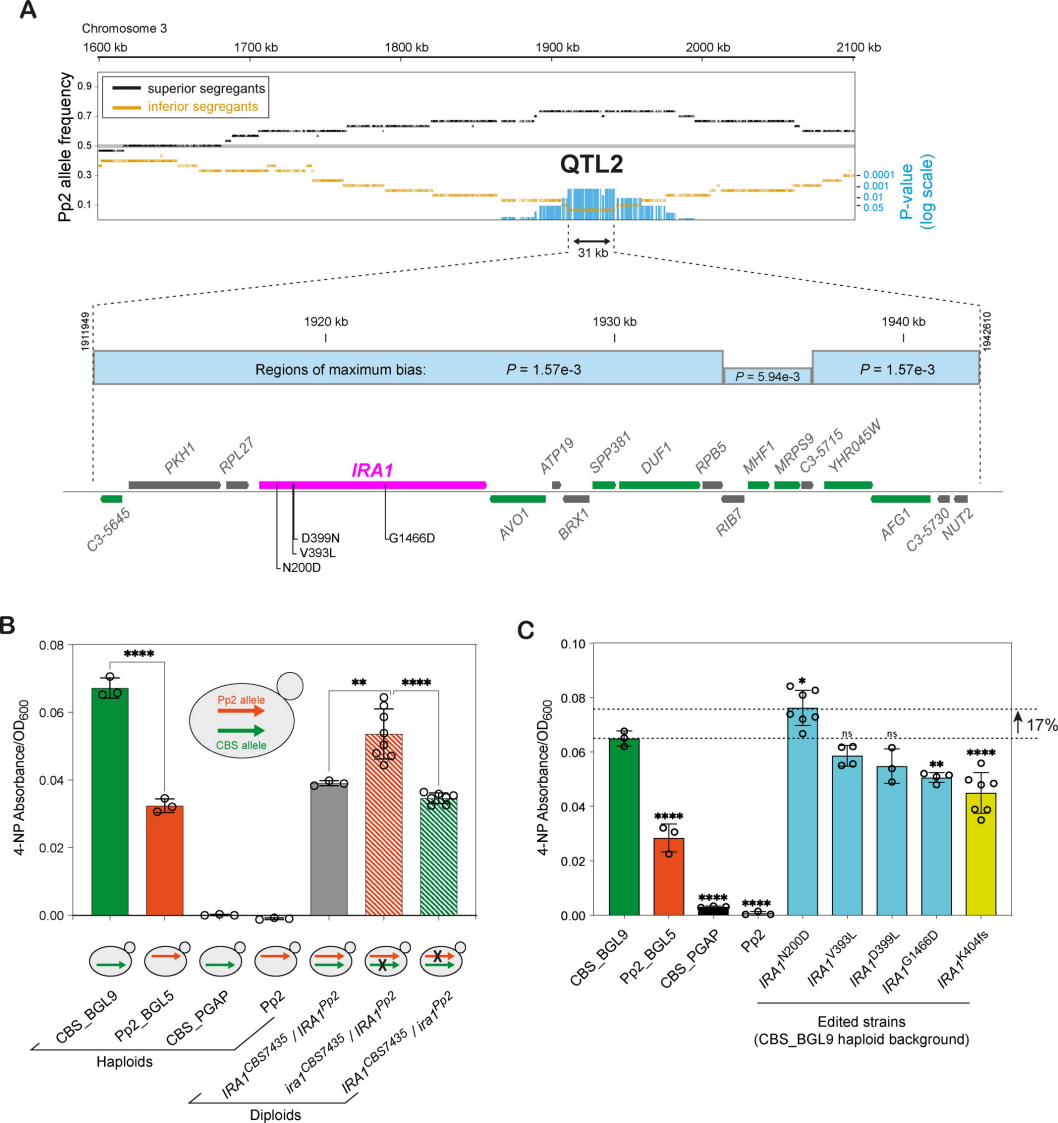
QTL2 maps to an N200D variant in *IRA1*. **(A**) Detailed map of the QTL2 region in Cross 1. The upper panel shows Pp2 allele frequencies in the QTL2 region on chromosome 3, among the 30 superior and 30 inferior segregants in Cross 1, as in Fig 3A. Blue vertical bars indicate *P* values for biased segregation of alleles at individual SNP sites. The peak of QTL2 is 31 kb long and consists of 2 regions of 22 kb and 7 kb, each with *P* = 1.57 × 10^−3^, separated by a 2-kb region with *P* = 5.94 × 10^−3^. The lower panel shows a gene map of the 31-kb interval. *IRA1* and the 8 genes colored green contain nonsynonymous SNPs in Pp2 relative to CBS7435 that are absent in Pp4. The 4 such SNP sites in *IRA1* are labeled. (**B**) Reciprocal hemizygosity analysis of the effect of *IRA1* alleles on BGL secretion in a CBS_BGL9/Pp2_BGL5 diploid. Haploid strains are included for comparison. X symbols in the cartoons indicate *IRA1* alleles disrupted by insertion of a *NatMX* antibiotic resistance marker. Bars represent an average of 4-NP absorbance values from 3 independent cultures of control strains and at least 7 biological replicates from the reciprocally hemizygote strains. Error bars indicate standard deviation. Significant differences in BGL secretion were tested with unpaired *t* tests (two-tailed) and are indicated by asterisks (**, *P* < 0.01 and ****, *P* < 0.0001). (**C**) Effects of *IRA1* SNP editing on BGL secretion. The edited strains were made in the haploid CBS_BGL9 background and contain individual nonsynonymous substitutions (N200D, V393L, D399N, G1466D) or a frameshift mutation (K404fs). Bars show mean 4-NP absorbance values from 3 independent cultures of control strains and at least 3 biological replicates from the edited strains. Error bars show standard deviation. Significant differences in BGL secretion between CBS_BGL9 and the other strains were tested by one-way ANOVA (Dunnet correction for multiple comparisons) and are indicated by asterisks (*, *P* < 0.05. **, *P* < 0.01 and ****, *P* < 0.0001) as well as ns (nonsignificant). Numerical data are listed in S1 Data.

At the peak of QTL2, the Pp2 allele is present in 22 (73%) of the 30 superior BGL secreting segregants, as compared to 2 (7%) of the 30 inferior segregants, in 2 neighboring regions (each with *P* = 1.57 × 10^−3^) in a 31-kb interval (Fig 5A). This interval contains 18 genes. Because QTL2 was detected in Cross 1 but not in Cross 2, we filtered the SNPs in these genes to exclude any variants that are shared by both Pp2 and Pp4 relative to CBS7435. Nine of the 18 genes contain at least 1 nonsynonymous SNP that passed this filter (Fig 5A). We reviewed the functions of these 9 genes and identified *K*. *phaffii IRA1* as a strong candidate gene, because in *S*. *cerevisiae*, a defect in *IRA2* was previously found to inhibit the degradation of aggregates of a misfolded heterologous secreted protein in the endoplasmic reticulum (ER) [43], and ER stress is a known bottleneck in protein secretion by *K*. *phaffii* [15]. Due to the whole-genome duplication in *S*. *cerevisiae*, *IRA1* of *K*. *phaffii* is orthologous to both *IRA1* and *IRA2* of *S*. *cerevisiae* [44].

To test whether alleles of *IRA1* affect BGL secretion, we first used reciprocal hemizygosity analysis in a CBS_BGL9/Pp2_BGL5 diploid background (Fig 5B). This diploid (*IRA1*^*CBS7435*^*/IRA1*^*Pp2*^) secretes BGL at a level similar to the lower of its 2 haploid parents, Pp2_BGL5, probably because the *hoc1*^*CBS7435*^ allele is recessive (Fig 4B). However, a hemizygous derivative of this diploid, in which the *IRA1*^*CBS7435*^ allele is disrupted and the *IRA1*^*Pp2*^ allele remains intact, shows significantly increased BGL secretion (Fig 5B). In contrast, the reciprocal hemizygote with a disruption of only the *IRA1*^*Pp2*^ allele shows little change in BGL secretion. These results functionally confirm that *IRA1*^*Pp2*^ is a recessive beneficial allele at QTL2 for improved BGL secretion.

### QTL2 is caused by an *IRA1*^N200D^ variant in Pp2

There are 6 amino acid differences between the proteins encoded by the *IRA1* alleles of Pp2 and CBS7435. Two of these differences are at sites where Pp2 and Pp4 have the same amino acid change relative to CBS7435, so they cannot be the cause of QTL2. We therefore focused on the other 4 sites (Fig 5A). We used CRISPR/Cas9 editing [42] to incorporate each of these 4 amino acid substitutions individually into the *IRA1* gene of the haploid CBS_BGL9 parental strain. Additionally, a CBS_BGL9 derivative with a frameshift mutation in *IRA1* (*IRA1*^K404fs^) was obtained fortuitously during CRISPR/Cas9 editing.

Phenotyping these edited strains showed that incorporating an *IRA1*^N200D^ substitution into haploid CBS_BGL9 significantly improved its BGL secretion, by an average of 17% in 4-day 400 μl cultures (Fig 5C). No improvement was seen in the strains harboring the other 3 nonsynonymous edits, most of which showed no significant difference in BGL secretion vis-à-vis CBS_BGL9. The strain with the frameshift showed significantly less BGL secretion than CBS_BGL9 (Fig 5C), suggesting that the N200D variant may increase Ira1 activity.

**Fig 6:**
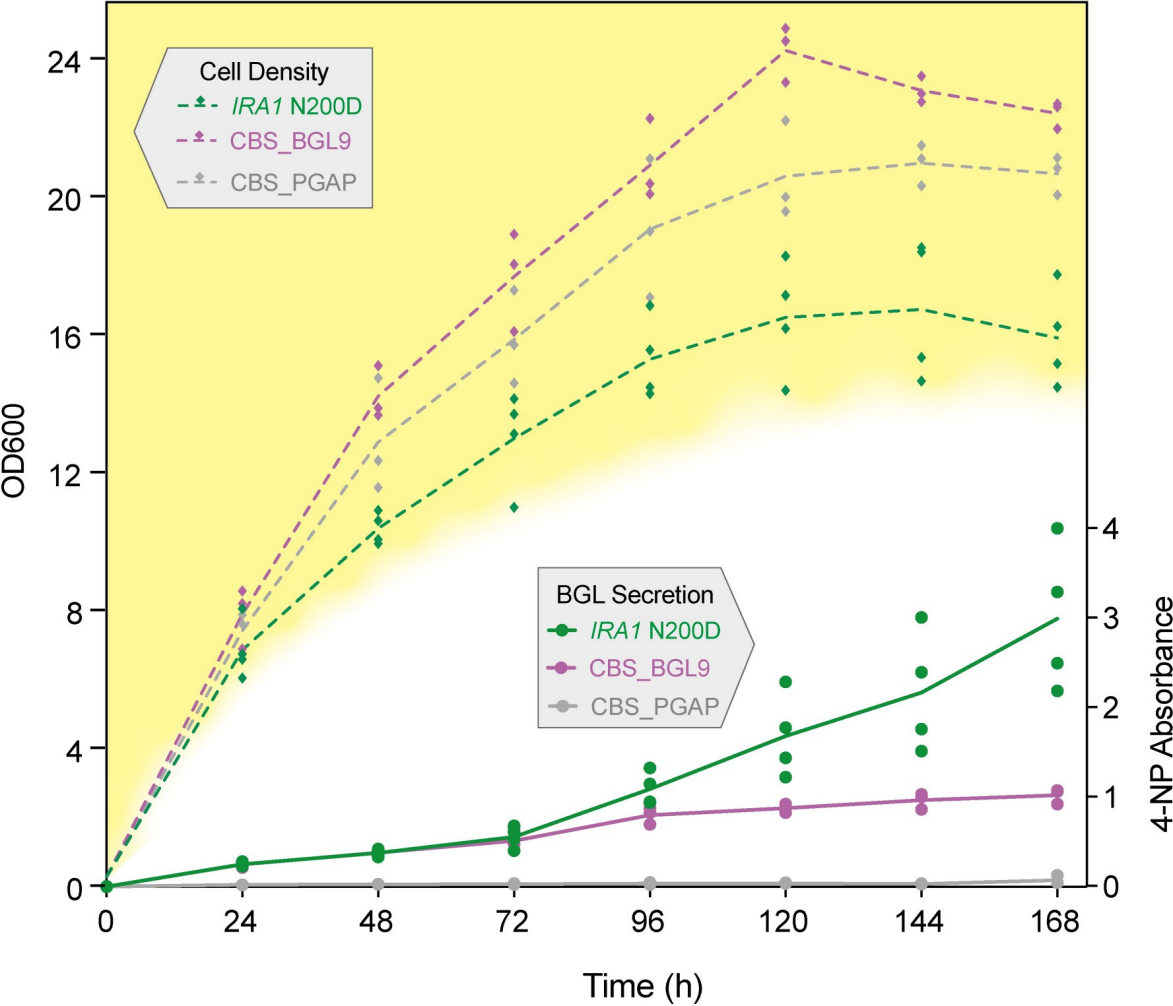
Effect of the *IRA1*^N200D^ substitution on BGL secretion in 1-week, 100-ml shake-flask cultures. BGL secretion and cell density data from 4 independently edited clones with the *IRA1*^N200D^ edit (green points) are compared to 3 independent cultures of their parent CBS_BGL9 (purple) and its empty vector counterpart CBS_PGAP (gray). Lines indicate trends in average 4-NP absorbance and OD_600_ with respect to time. Numerical data are listed in S1 Data.

To further test the effect of the *IRA1*^N200D^ substitution, we measured BGL secretion and growth rate in 100-ml shake-flask cultures over a 1-week period. We compared 4 independently edited clones of CBS_BGL9 harboring the *IRA1*^N200D^ edit to the unedited parental strain CBS_BGL9 and an empty vector control strain (CBS_PGAP), assaying BGL secretion every 24 hours. The *IRA1*^N200D^ clones grew more slowly than their unedited parent and secreted more BGL at every time point from 96 hours onward (Fig 6). By the end of the experiment (168 hours), the *IRA1*^N200D^ clones had secreted 2.96 times the amount of BGL secreted by their unedited parent. Due to the slower growth of the *IRA1*^N200D^ clones, normalizing BGL secretion by cell density shows that they reached 3.8 times the parental level per cell at 168 hours (S6 Fig).

Since most BGL production is seen in the late growth phase and the *IRA1*^*N200D*^*-*edited clones have slower growth rates than unedited clones (Fig 6), we examined cell viability and the possibility that the excess BGL production in the edited clones is caused by cell lysis rather than secretion. SDS-PAGE analysis of supernatants from these cultures confirms that BGL is secreted as a relatively pure protein at the expected size of 120 kDa (S7 Fig). Consistent with the results in Fig 6, the intensity of the BGL band is higher in the *IRA1*^*N200D*^*-*edited clones than in unedited controls, and this difference becomes more pronounced as time progresses from 96 hours to 168 hours (S7 Fig). Propidium iodide staining shows that cell viability in the edited clones decreases to 40% to 50% in the edited clones from 72 hours onward, whereas it decreases little in unedited clones (S8 Fig). Cell lysis, visible as additional bands on the SDS-PAGE gels, is apparent in the *IRA1*^*N200D*^*-*edited clones, but only at the final 168-hour time point (S7 Fig).

### *IRA1*^N200D^ does not improve secretion of α- or β-galactosidases

To test whether the *IRA1*^N200D^ variant has a positive impact on secretion of other heterologous proteins, we introduced it by CRISPR/Cas9 editing into 2 haploid *K*. *phaffii* strains with CBS7435 genetic backgrounds that express codon-optimized *Aspergillus niger* α-galactosidase (strain IT1018) or β-galactosidase (strain IT1005) from the constitutive promoter UPP [45]. We then used enzyme activity assays to compare galactosidase secretion every 24 hours between multiple edited and unedited clones of these strains in 400 μL cultures grown for 96 hours. The *IRA1*^N200D^ variant had no consistent effect on secretion of either of the galactosidase proteins (S9 Fig), so its effect on BGL secretion appears to be specific to that protein.

## Discussion

We identified variants of 2 genes, *HOC1* and *IRA1*, that significantly influence secretion of a test protein in *K*. *phaffii*. At the *HOC1* locus (QTL1), CBS7435 has a frameshifted (presumably null) allele that gives a superior secretion phenotype. This *hoc1*^CBS7435^ allele must have arisen during the development of the CBS7435 strain by Phillips Petroleum in the 1970s [5,46] because the frameshift is not present in NRRL Y-7556, the natural isolate from which CBS7435 was derived, even though the genomes of these 2 strains are almost identical [30,31]. Brady and colleagues [31] reported that 4 *K*. *phaffii* strains (including NRRL Y-7556, Pp2, and Pp4) have thick cell walls and are resistant to the cell wall-perturbing drug Calcofluor White, whereas 7 others (CBS7435 and its derivatives including GS115 and X-33) have thinner walls and are sensitive. Our results show that the thick cell wall phenotype is due to the presence of an intact *HOC1* gene and that the frameshifted *hoc1*^CBS7435^ allele results in both a thinner cell wall and higher levels of heterologous protein secretion. Brady and colleagues [31] also found that, during growth on methanol media, CBS7435 and the other thin-walled strains had higher levels of methanol uptake and higher expression of methanol utilization pathway genes than the thick-walled strains. The *hoc1*^CBS7435^ frameshift may therefore have arisen during selection by Phillips Petroleum for strains showing increased methanol utilization, in efforts to convert methanol into single-cell protein for use as animal feed [46,47], and its cell wall defect subsequently turned out to be beneficial for heterologous protein secretion.

At the *IRA1* locus (QTL2), *K*. *phaffii* strain Pp2 has an allele that gives a superior secretion phenotype at the cost of lower cell viability, which we mapped to the amino acid substitution N200D. However, this substitution did not improve secretion of α- or β-galactosidase, so its effect appears to be specific to BGL and not broadly transferrable to other heterologous proteins. *S*. *cerevisiae* Ira1 and Ira2 are negative regulators of the Ras/PKA pathway, which is the main pathway for carbon nutrient signaling [48,49]. The N200D site is near the N-terminus of Ira1, in a region that is conserved among fungi but not in other eukaryotes (S10 Fig). This region is not near the GTPase activator domain that interacts with Ras, and its function is unknown.

To our knowledge, this study is the first QTL analysis in *K*. *phaffii*. We combined phenotyping a large number of segregants (1,000 per cross, to select segregants with extreme phenotypes) with sequencing the genomes of the selected segregants individually (30 per pool) instead of in bulk. We found that the latter step dramatically reduced noise in the QTL signal (compare Figs 3 and S4). We also found that the *K*. *phaffii* QTL peaks are broader than those usually seen in *S*. *cerevisiae*, which is a consequence of its 4-fold lower number of crossovers per meiosis [30]. One way to make the peaks sharper would be to conduct multiple cycles of intercrossing [50].

Our work demonstrates the feasibility of using QTL analysis to find genes underlying phenotypes of biotechnological interest in nonconventional yeasts. We have shown that natural isolates can contain beneficial alleles that are absent from the most widely used strains of these species, but also that alleles beneficial to the secretion of 1 heterologous protein may not be beneficial to others. Because research on other nonconventional yeasts is often centered on a single genetic background, it is likely that QTL approaches will be of value in these species too.

## Materials and methods

### Experimental design

We used the approach of sequencing selected segregants with extreme phenotypes [51] because we were investigating only a single phenotype. We defined the superior and inferior pools as the top and bottom 3% of the phenotypic distribution, in line with previous *S*. *cerevisiae* studies [20,21] and theoretical modeling [51]. We phenotyped 1,000 segregants per cross because this was the largest number we could handle. We sequenced the selected segregants individually because we expected that it would increase resolution. For choosing candidate genes in QTL regions, we relied primarily on the *S*. *cerevisiae* literature and information about gene functions in the *Saccharomyces* Genome Database [52] and looked for genes with connections to the secretory pathway or ER. In the QTL1 region, we initially investigated and eliminated 4 other candidate genes (*TRL1*, *VAC7*, *HOS4*, and *COG5*) before we noticed that the *HOC1* gene in the reference *K*. *phaffii* genome sequence was misannotated and contained a frameshift relative to other strains. In the QTL2 region, we investigated only *IRA1* because no other genes in the region had literature connections to secretion.

### Strains, media, and general molecular biology methods

Yeast strains used in this study are listed in S1 Table. Strains were cultured at 30°C in premixed YPD medium (10 g/L yeast extract, 20 g/L bacteriological peptone, and 20 g/L glucose; Formedium, UK) with shaking at 200 rpm for liquid cultures. Solid YPD nutrient plates had a supplement of 15 g/L bacto agar (Formedium, UK).

Diagnostic PCR amplifications were performed using GoTaq Green Master Mix (Promega) or, for colony screening, Phire Green Hot Start II PCR Master Mix and Phire Plant Direct PCR Master Mix (Thermo Fisher Scientific). High-fidelity PCR amplifications for cloning or Sanger sequencing were performed with Q5 DNA polymerase (New England Biolabs). All PCR amplifications were performed following the DNA Polymerase manufacturer’s recommendations. When required, PCR amplicons were purified using the QIAquick PCR Purification Kit or the QIAquick Gel Extraction Kit (Qiagen). Cloning reaction mixtures were transformed into competent DH5α *E*. *coli* and selected on LB agar media containing antibiotics (25 μg/mL zeocin, 50 μg/mL kanamycin, 50 μg/mL nourseothricin, or 50 μg/mL hygromycin B). Plasmid minipreps were performed with the QIAprep Spin Miniprep Kit (Qiagen).

The coding sequence of the *Thermoascus aurantiacus BGL* (*bgl1*) gene [35] (GenBank accession number DQ114397.1) was synthesized as a gBlock gene fragment (Integrated DNA Technologies) and inserted in-frame with the *S*. *cerevisiae* α-factor secretion signal on the pGAPZα plasmid (Invitrogen) by Gibson assembly. To generate a construct conferring geneticin (G418) resistance in *K*. *phaffii*, the zeocin resistance gene (*ZeoR*) in the pGAPZα-BGL construct was replaced with a kanamycin/geneticin resistance gene (*KanR*) by Gibson assembly. Linear BGL expression cassettes were generated from these circular constructs by cutting them within the *GAP* promoter using *Avr*II (New England Biolabs). Transformation of *K*. *phaffii* with linear BGL expression cassettes was achieved by electroporation (Bio-Rad Laboratories Gene Pulser II) as described [53]. Transformants were selected on zeocin (100 μg/mL) or geneticin (500 μg/mL) and screened by PCR to check correct integration of the BGL expression cassettes at the *GAP* locus using forward and reverse primers flanking the targeted *GAP* promoter. The structure and single-copy nature of the BGL expression cassette was confirmed by genome sequencing (Illumina) of the 3 clones used as parents for crosses (CBS_BGL9, Pp2_BGL5, and Pp4_BGL3).

### Mating, sporulation, and random spore isolation

Crosses were carried out by velvet replica plating onto NaKG mating plates (0.5% sodium acetate, 1% potassium chloride, 1% glucose, 2% bacto agar) [36,54]. The mating plate was incubated for 2 days at room temperature and subsequently replica plated onto diploid selection medium (YPD supplemented with 200 μg/mL zeocin and 1 mg/mL geneticin). Diploids were incubated for 3 days at 30°C and streaked for a second time for phenotype confirmation and generation of single colonies for cryopreserved stock. Sporulation was induced by streaking diploid cells on NaKG plates and incubating for 10 to 20 days at room temperature. A loop of sporulated material was resuspended in 1 mL sterile distilled water to an OD_600_ ~2. Vegetative cells were killed by adding 1 mL diethyl ether to the cell suspension and incubating the mixture for 15 minutes on a roller. Diethyl ether was removed, and the spore suspension was diluted in series up to 10^−4^. Dilutions of 10^−3^ and 10^−4^ were plated on YPD plates for 2 to 3 days at 30°C until colonies were visible. These colonies, which result mostly from individual spores, were picked individually and patched on a fresh YPD plate and incubated at 30°C overnight, for subsequent replica plating onto a set of diagnostic plates for selection of the 2 spore types: *ZeoR* (zeocin 200 μg/mL) or *KanR* (geneticin 1 mg/mL). A third plate with both antibiotics was used to identify and discard any colonies that could have arisen from a mixture of spores, given the propensity of *K*. *phaffii* spores to clump together [36].

### Phenotypic evaluation of BGL secretion

Strains were pre-cultured in YPD at 30°C for 48 hours, either as 400 μL cultures in deep-well plates with agitation at 1,100 rpm, or as 1 ml cultures in test tubes with agitation at 250 rpm. Strains harboring plasmids (e.g., for CRISPR/Cas9 genome editing) were cured of the plasmids before preculturing. Approximately 5 μL of precultured strains was used to inoculate 600 μL YPD in deep-well plates and incubated in a final volume of 400 μL (30°C, 1,100 rpm, 96 hours), after withdrawing 200 μL from each well to measure initial OD_600_. Following the 96-hour incubation, plates were spun immediately (4,000 rpm, 5 minutes, 4°C) followed by aliquot withdrawals of the supernatants from each strain for storage (−20°C) or placed on ice for immediate analysis of BGL secretion. To analyze BGL secretion from strains, 2 μL supernatants or appropriate dilutions thereof were mixed with 98 μL of 5 mM solution of 4-NPG (Sigma) in sodium acetate buffer (0.1 M, pH 5) in 96-well plates. Replicates of 2 μL YPD in 98 μL 4-NPG were used as blanks. Plates were incubated at 40°C for 10 minutes followed by the addition of 100 μL, 1 M Na_2_CO_3_. 4-NP absorbance was subsequently determined by reading plates at 405 nm in a plate reader (Molecular Devices Spectramax190 for Cross 1 segregants, or BioTek Synergy H1 for Cross 2 segregants). Actual 4-NP absorbance for each sample was determined after deducting mean blank absorbance values.

### Galactosidase enzyme assays

Galactosidase enzyme activity assays were performed by following the same steps as described for BGL activity assays with the exception of substrates used and incubation temperature for enzyme-substrate reactions. The substrates were 5 mM O-Nitrophenyl-beta-D-galactopyranoside (ONPG) (Sigma) and 2 mM p-Nitrophenyl-alpha-D-galactopyranosidase (pNPG) (Sigma) to assay β- and α- galactosidase activities, respectively. Supernatant (enzyme)-substrate reactions were incubated at 50°C for 10 minutes.

### SDS-PAGE

Samples for SDS-PAGE analysis were processed by mixing 34.5 μL of supernatants with 11.5 μL of 4X Bolt LDS protein loading buffer (Thermo Fisher Scientific) followed by boiling at 99°C for 10 minutes. Boiled samples were briefly centrifuged (1 minute, 13,000 rpm), after which 40 μL of each were loaded onto Bolt 8% Bis-Tris Plus precast gels (Thermo Fisher Scientific) and run alongside the PageRuler Plus Prestained Protein Ladder in a Mini Gel Tank (Thermo Fisher Scientific). The run was performed at 150 V for 35 minutes with 1X MES SDS running buffer (Thermo Fisher Scientific). Gels were stained with instant blue dye (Expedeon) for 1 hour before imaging.

### Ploidy and cell viability measurement by flow cytometry

Ploidy was estimated with a modified version of the method of Popolo and colleagues [55]. Briefly, cells patched on YPD overnight were suspended in 1 mL sterile ice-cold water to OD600 ~1, centrifuged (5 minutes, 5,000 rpm), and fixed in 1 mL cold 70% ethanol for 24 hours at 4°C (to minimize cell aggregation during ethanol fixation, cells were first resuspended in 300 μL cold sterile water followed by dropwise addition of 700 μL cold absolute ethanol while vortexing). Fixed cells were next treated with 100 μL 1 mg/mL RNAse A for 90 minutes at 37°C after centrifugation to remove the ethanol. RNAse A–treated cells were centrifuged and the pellet stained with 100 μL 0.05 mg/mL propidium iodide at 4°C for 24 hours. For cell viability assays, cells were washed once with PBS by centrifuging at low speed (5 minutes, 5,000 rpm), stained directly with the abovementioned volume and concentration of propidium iodide in PBS. Stained samples were incubated for 30 minutes at room temperature before flow cytometry. Approximately 50 μL of stained cells was diluted to 500 μL with ice-cold water and run on a Becton Dickinson Accuri C6 flow cytometer during ploidy determination and on the CytoFLEX LX (Beckman Coulter) during viability assays on slow fluidics. Data were analyzed using Flowjo software (Flowjo, LLC).

### Genomic DNA isolation and whole genome sequencing

Genomic DNA was extracted from 10 mL stationary-phase cultures of individual segregants by homogenization with glass beads followed by phenol-chloroform extraction and ethanol precipitation. All 120 individual segregants and the 4 pools of segregants were sequenced to approximately 100× coverage (paired-end, 2 × 150 bp reads) on an Illumina HiSeq 4000 instrument (BGI Tech Solutions, Hong Kong).

### Bioinformatics

The Burrows-Wheeler Aligner [56] (BWA version 0.7.9a; with parameters -M and -Y) was used to generate SAM alignments of the Illumina reads from each segregant to a reference genome sequence of *K*. *phaffii* strain CBS7435 (nuclear chromosomes and cytosolic linear plasmids from Sturmberger and colleagues [29], accession numbers LT962476.1 to LT962479.1, MG491503.1 and MG491504.1; mitochondrial genome from Kuberl and colleagues [57], accession number FR839632.1). Sorted and indexed BAM alignments were then created using SAMtools (version 0.1.19). Read coverage for each segregant was checked using Bedtools (version 2.18.1) to rule out any instances of aneuploidy or CNV. Duplicates were then identified and a deduplicated BAM alignment created using Picard Tools (version 2.0.1). Variants against our reference were called with the GATK (version 4.1.1.0) HaplotypeCaller tool with the “emitRefConfidence GVCF” parameter. These GVCF files were then combined using GATK (“CombineGVCFs”) into joint genotype calls across all the segregants and outputted as a multisample VCF file of all variant sites (“GenotypeGVCFs”), and for downstream analysis converted to a table with the genotype information (“VariantsToTable”).

Perl scripts were used for parsing and reformatting data. Any variants that were not called in all segregants, were multi-nucleotide, and/or had a spanning deletion or had artifactual heterozygous calls were filtered out, and only SNPs present in the Pp2 or Pp4 parent were used in the analysis of that cross. After this filtering, a matrix was created for each cross of the joint genotype calls (CBS7435 = REF = 1; Pp2/Pp4 = ALT = 0) across all SNPs (rows, ordered by chromosomal position) for all segregants (columns, ordered by BGL secretion level), and these were the basis for most subsequent analyses. The numbers of nuclear SNPs available for QTL analysis were 42,262 in Cross 1 (Pp2_BGL5 × CBS_BGL9), and 41,552 in Cross 2 (Pp4_BGL3 × CBS_BGL9). All SNPs were given unique names, with a leading letter to represent its chromosome (lowercase for Pp2; uppercase for Pp4) and a 5-digit number for its order on that chromosome.

For each SNP, Fisher’s exact test (implemented in R version 3.2.1) was used to identify significant differences in frequency of the Pp2/Pp4 allele between the 30 superior and 30 inferior segregants. Given the large number of SNPs on each chromosome the resulting *P* values (blue y-axis in Fig 3) were corrected for multiple testing with a Bonferroni correction applied for each chromosome.

The allele frequency and *P* value data at each SNP site in the top 30 and bottom 30 segregants in each cross (Fig 3) are provided as S2 Data. The data matrix files of genotypes in each segregant are provided as S3 and S4 Data.

### Reciprocal hemizygosity analysis

Reciprocally hemizygous strains were created by disrupting *IRA1* alleles by integrating a plasmid containing *NatMX* cassette and an internal fragment of *IRA1* via a single crossover. The plasmid was assembled using the NEBuilder HiFi DNA Assembly Master Mix (New England Biolabs). PCR with primers binding within the *IRA1* fragment was used to linearize the plasmid for transformation. The linear cassette was transformed into the CBS_BGL9/Pp2_BGL5 diploid by electroporation followed by selection on YPD plates containing 100 μg/mL nourseothricin. Transformants were screened for correct integration of the disruption cassette by colony PCR amplification of the upstream and downstream junctions flanking the disruption site. These amplicons were sequenced, and SNPs both upstream and downstream of the disruption site were used to identify the parental origin of the disrupted allele in each transformant. The presence of an intact second *IRA1* allele was confirmed by PCR using primers binding outside the internal recombination fragment.

### CRISPR/Cas9-mediated genome editing

Details of guide RNA (gRNA) targets and repair templates used in CRISPR/Cas9 editing are given in S2 Table. Editing of SNPs was carried out by the method of Gassler and colleagues [42], using the CRISPi kit they developed (Addgene Kit #1000000136). gRNA targets were chosen to be in close proximity to SNPs targeted for editing and cloned in the BB3cH_pGAP_23*_pLAT1_Cas9 plasmid from the kit by Golden Gate cloning, following the authors’ recommendations. Transformants were selected on 250 μg/ml hygromycin B. A single gRNA was used to edit *IRA1* SNPs V393L and D399N because they are in close proximity. Repair templates were made by overlap-extension PCR, incorporating the required SNP changes into the primers used to generate the overlap, and (for *IRA1* SNPs N200D and G1466D) by amplification of one part of the repair template from Pp2 genomic DNA. For all SNP edits, the PAM site was altered by making a synonymous change within the overlap region of the repair template to prevent recutting by Cas9. Editing was confirmed by Sanger sequencing.

### Spot assays on Calcofluor White

Spot assays were performed following previously described methods [31,58]. Strains were grown overnight in YPD broth and washed twice in sterile double-distilled water. Resulting pellets were resuspended in 700 μL sterile double-distilled water and diluted to OD_600_ = 0.5, followed by 10-fold serial dilutions in 96-well microtiter plates until a final dilution of 10^−4^. Approximately 5 μL of each dilution series were spotted onto YPD plates and onto YPD supplemented with Calcofluor White (Sigma) to a final concentration of 20 μg/mL. Plates were incubated at 30°C for 2 days.

## Supporting information

S1 Fig Expression constructs and assessment of extracellular BGL secretion. **(A)** Maps of the 2 BGL expression cassettes bearing zeocin (*ZeoR*) and geneticin (*KanR*) resistance markers. Also shown is the position and orientation of the cloned *T*. *aurantiacus BGL* gene. α-F, *S*. *cerevisiae* alpha-factor secretion signal. (**B)** UV imaging for qualitative assessment of extracellular BGL secretion in transformed clones of CBS7435, Pp2, and Pp4 using 4-MUG assays in 24-well microtiter plates. UV fluorescence occurs due to BGL hydrolysis of 4-MUG to 4-methylumbelliferone. (**C)** Line graphs showing quantitative time point assessment of BGL secretion in transformed clones of CBS7435, Pp2, and Pp4 using 4-NPG assays (3 ml cultures incubated for 96 hours). Optical absorbance at 405 nm occurs due to BGL hydrolysis of 4-NPG to 4-NP. Clones selected for use as parents in genetic crosses are marked with black rectangles. Numerical data are listed in S1 Data. BGL, β-glucosidase; 4-MUG, 4-methylumbelliferyl-β-D-glucuronide; 4-NP, 4-nitrophenol; 4-NPG, 4-nitrophenol-β-D-glucopyranoside.
(PDF)

S2 Fig Flow cytometric assessments of ploidy. **(A**) Parental *K*. *phaffii* strains and diploids formed by mating. AMY429 and BY4742 are *S*. *cerevisiae* diploid and haploid strains, respectively, included as controls. Dashed red lines indicate haploid *K*. *phaffii* cell populations in the G1 and G2 phases of the cell cycle. (**B, C)** Ploidy screening of (**B**) superior segregants and (**C**) inferior segregants, from Cross 1. The top 2 histograms in each group show the DNA content of the diploid (CBS_BGL9/Pp2_BGL5, light red) and 1 haploid parent (Pp2_BGL5, blue) for comparison as diploid and haploid controls, respectively. Segregants that were not included in the final pools of 30 segregants are named in parentheses (segregants were excluded if they had diploid profiles, inconsistent rephenotyping results, or were simply supernumerary). (**D, E)** Ploidy screening of (**D**) superior segregants and (**E**) inferior segregants, from Cross 2. The top 2 histograms in each group show the DNA content of the diploid (CBS_BGL9/Pp4_BGL3, blue) and 1 haploid parent (Pp4_BGL3, red) for comparison as diploid and haploid controls, respectively. Segregants that were not included in the final pools of 30 segregants are named in parentheses. Raw data (FCS files) have been deposited in FlowRepository (https://flowrepository.org/).
(PDF)

S3 Fig Phenotypes of segregants. **(A, B)** Distributions of BGL activity among all segregants in **(A**) Cross 1 and (**B**) Cross 2. The green lines show best-fit normal distribution curves. **(C, D**) Comparison of normalized 4-NP absorbance (BGL secretion) measurements in initial phenotyping and rephenotyping of segregants in (**C**) Cross 1 and (**D**) Cross 2. Red and green points denote inferior and superior segregants, respectively. **(E, F**) Comparison of normalized cell density (OD_600_) measurements in initial phenotyping and rephenotyping of segregants in (**E**) Cross 1 and (**F**) Cross 2. Red and green points denote inferior and superior segregants, respectively. Numerical data are listed in S1 Data.
(PDF)

S4 Fig Plots of SNP frequency versus chromosomal position in pooled (bulk) segregant analysis. **(A)** Pp2 allele frequencies in Cross 1. Dot plots show the location and estimated allele frequencies, obtained by bulk segregant sequencing, of Pp2 SNP alleles along the 4 chromosomes of *K*. *phaffii*. Green dots indicate the frequency of Pp2 SNP alleles in Illumina reads from the DNA pool of 30 superior segregants, whereas red dots indicate their frequency in the DNA pool of 30 inferior segregants. Broken magenta lines indicate the 50% allele frequency expected for loci unlinked to the BGL secretion phenotype. (**B**) Pp4 allele frequencies in Cross 2. Dot plots show the location and estimated allele frequencies, obtained by bulk segregant sequencing, of Pp4 SNP alleles along the 4 chromosomes of *K*. *phaffii*. Green dots indicate the frequency of Pp4 alleles in Illumina reads from the DNA pool of 30 superior segregants, whereas red dots indicate their frequency in the DNA pool of 30 inferior segregants. Numerical data are listed in S1 Data.
(PDF)

S5 Fig *hoc1* mutants are sensitive to Calcofluor White. Serial dilution spot test assays were used to compare the susceptibility to Calcofluor White (CFW, 20 μg/mL) of haploid *K*. *phaffii* strains with wild-type *HOC1* and mutant *hoc1* genes. (**A**) Unedited parental strains. (**B**) Edited strains. Genotypes at the *HOC1* locus are indicated on the right.
(PDF)

S6 Fig Time course comparing OD_600_-normalized BGL secretion between CBS_BGL9 and its derivatives containing the *IRA1*^N200D^ edit. The data from the experiment in Fig 6 were reanalyzed to normalize BGL secretion (4-NP absorbance) by cell density (OD_600_) in each culture. Numerical data are listed in S1 Data.
(PDF)

S7 Fig SDS-PAGE analysis of BGL secretion over time in clones with and without *IRA1*^N200D^. Proteins were extracted from supernatants of 100-ml shake-flask cultures at 96 hours, 120 hours, 144 hours, and 168 hours. Comparisons are between CBS_PGAP (3 technical replicates), CBS_BGL9 (3 technical replicates), and ICs (4 independently clones in which the *IRA1*^N200D^ variant was introduced by genome editing). The arrow indicates BGL, which migrates at its expected size of 120 kDa. L, molecular size standards. Due to a problem during gel loading, the sample from the fourth IC at 144 hours was split between 2 lanes. The cultures used for this experiment were the same ones used in Fig 6.
(PDF)

S8 Fig Time-course assessment of cell viabilities during BGL secretion. **(A)** Percentage viabilities indicate the proportion of cells that were determined by flow cytometry as exhibiting minimal intracellular accumulation of propidium iodide. Purple and gray points denote cell viability measurements in 3 independent cultures of CBS_BGL9 and CBS_pGAP, respectively. Green points denote 4 independently edited clones (ICs) in which the *IRA1*^N200D^ substitution was introduced into the CBS_BGL9 background by genome editing. **(B)** Cell density values (OD_600_) determined at the same timepoints as in (**A).** These measurements were made on a set of 1-week 100 ml shake-flask cultures that replicated the conditions used in Fig 6. Numerical data are listed in S1 Data.
(PDF)

S9 Fig Effect of the *IRA1*^N200D^ variant on secretion of galactosidase enzymes. **(A)** β-galactosidase activity of clones of IT1005 incorporating the *IRA1*^N200D^ edit (green dots, 7 independently edited clones) is compared to unedited clones from the same strain (orange squares, 2 independent clones) that were transformed for CRISPR editing but failed to incorporate the *IRA1*^N200D^ SNP, and to the original unedited strain IT1005 (blue triangles, 7 technical replicates). The negative control strains are CBS_pGAP (open inverted triangles, 7 technical replicates) and its *IRA1*^N200D^ derivative (brown diamonds, 3 technical replicates). **(B)** α-galactosidase activity of clones of IT1018 incorporating the *IRA1*^N200D^ edit (green dots, 11 independently edited clones) is compared to unedited clones from the same strain (orange squares, 2 independent clones) that were transformed for CRISPR editing but failed to incorporate the *IRA1*^N200D^ SNP, and to the original unedited strain IT1018 (blue triangles, 7 technical replicates). The negative controls are the same as in (**A)**. Numerical data are listed in S1 Data.
(PDF)

S10 Fig Conservation of the Asn200 site in Ira1/2 proteins of fungi. A section of a protein sequence alignment around position 200 of Ira1/2 proteins is shown. The arrow indicates the conserved asparagine (N) at position 200, which is substituted to aspartic acid (D) in *K*. *phaffii* isolate Pp2. This site corresponds to Asn226 of *S*. *cerevisiae* Ira1, and Asn253 of *S*. *cerevisiae* Ira2. The alignment was made using MUSCLE as implemented in Seaview v5.04 [59]. Colors indicate conservative amino acid groups. Sequence identifiers from MGOB [44] or NCBI are shown on the right.
(PDF)

S1 Table *Komagataella phaffii* strains used in this study. (PDF)

S2 Table Sequences of guide RNA targets, and primers used to amplify Repair Templates (RTs), for CRISPR/Cas9 editing of *HOC1* and *IRA1* genes. (PDF)

S1 Data Names of the segregants in each selected pool, and numerical data graphed in Figs 1, 2, 4, 5 and 6 and in S1, S3, S4, S6, S8 and S9 Figs. (XLSX)

S2 Data Allele frequencies at each SNP site in the selected segregant pools in Cross 1 and Cross 2. (XLSX)

S3 Data Genotypes of individual sequenced segregants from Cross 1. (TSV)

S4 Data Genotypes of individual sequenced segregants from Cross 2. (TSV)

S1 Raw Images Uncropped version of S7 Fig. (PDF)

## Abbreviations

BGL: β-glucosidase
ER: endoplasmic reticulum
gRNA: guide RNA
ONPG: O-Nitrophenyl-beta-D-galactopyranoside
pNPG: p-Nitrophenyl-alpha-D-galactopyranosidase
QTL: quantitative trait locus
SNP: single-nucleotide polymorphism
4-MUG: 4-methylumbelliferyl-β-D-glucuronide
4-NP: 4-nitrophenol
4-NPG: 4-nitrophenol-β-D-glucopyranoside
