# Supplementary material for: Identification of genetic variants of the industrial yeast Komagataella phaffii (Pichia pastoris) that contribute to increased yields of secreted heterologous proteins
Source: PLoS Biol. 2022 Dec 15;20(12):e3001877. doi: 10.1371/journal.pbio.3001877 (PMC9754263; doi:10.1371/journal.pbio.3001877)
Supplement: S1 Fig — (A) Maps of the 2 BGL expression cassettes bearing zeocin (ZeoR) and geneticin (KanR) resistance markers. Also shown is the position and orientation of the cloned T. aurantiacus BGL gene. α-F, S. cerevisiae alpha-factor secretion signal. (B) UV imaging for qualitative assessment of extracellular BGL secretion in transformed clones of CBS7435, Pp2, and Pp4 using 4-MUG assays in 24-well microtiter plates. UV fluorescence occurs due to BGL hydrolysis of 4-MUG to 4-methylumbelliferone. (C) Line graphs showing quantitative time point assessment of BGL secretion in transformed clones of CBS7435, Pp2, and Pp4 using 4-NPG assays (3 ml cultures incubated for 96 hours). Optical absorbance at 405 nm occurs due to BGL hydrolysis of 4-NPG to 4-NP. Clones selected for use as parents in genetic crosses are marked with black rectangles. Numerical data are listed in S1 Data. BGL, β-glucosidase; 4-MUG, 4-methylumbelliferyl-β-D-glucuronide; 4-NP, 4-nitrophenol; 4-NPG, 4-nitrophenol-β-D-glucopyranoside. (PDF) [file pbio.3001877.s001.pdf]

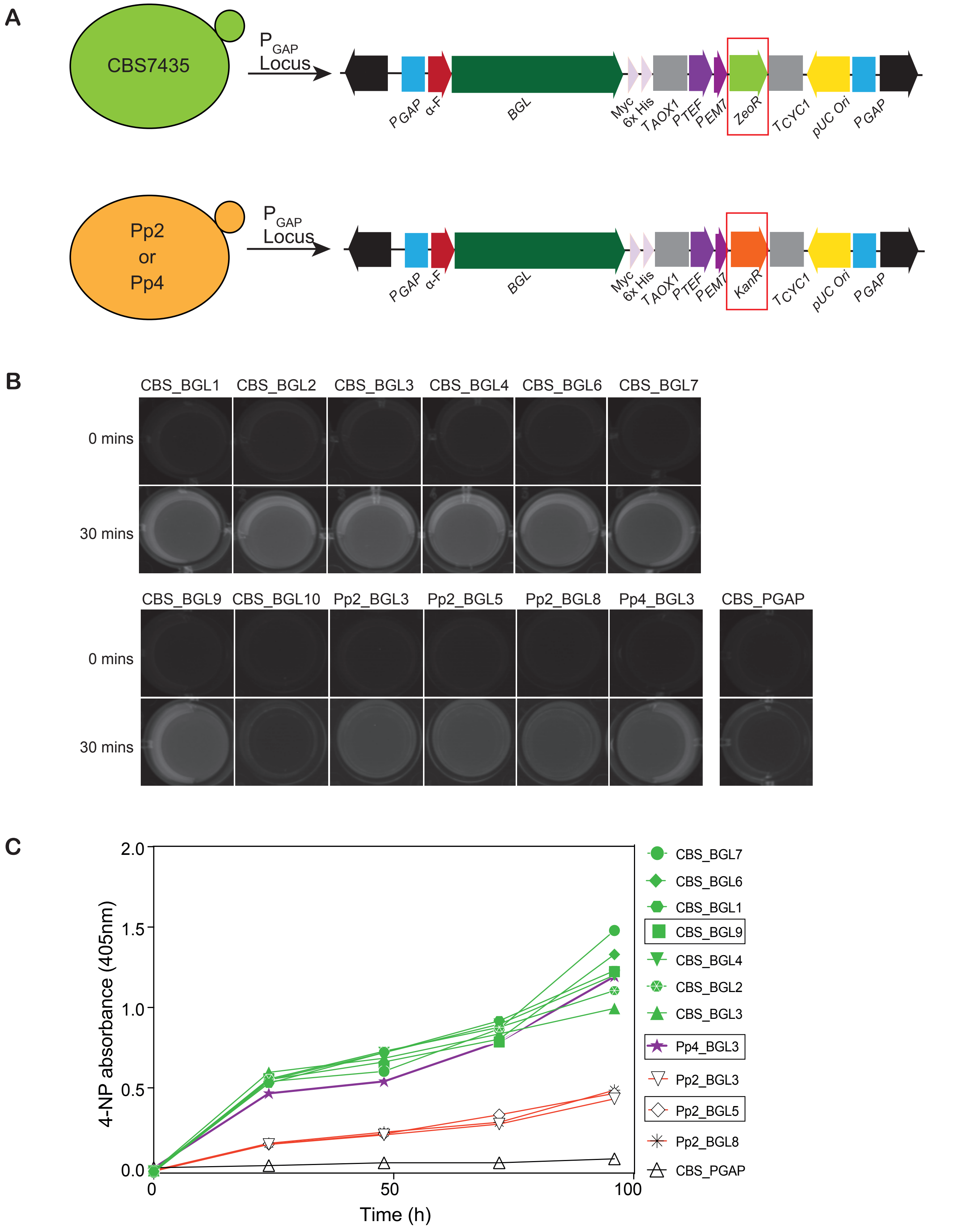

S1 Fig. Expression constructs and assessment of extracellular BGL secretion.

A, Maps of the two BGL expression cassettes bearing zeocin (*ZeoR*) and geneticin (*KanR*) resistance markers. Also shown is the position and orientation of the cloned *T. aurantiacus* β-glucosidase (*BGL*) gene. α-F, *S. cerevisiae* alpha-factor secretion signal.

B, UV imaging for qualitative assessment of extracellular BGL secretion in transformed clones of CBS7435, Pp2 and Pp4 using 4-MUG assays in 24-well microtitre plates. UV fluorescence occurs due to BGL hydrolysis of 4-methylumbelliferyl-β-D-glucuronide (4-MUG) to 4-methylumbelliferone.

C, Line graphs showing quantitative timepoint assessment of BGL secretion in transformed clones of CBS7435, Pp2 and Pp4 using 4-NPG assays (3 ml cultures incubated for 96 h). Optical absorbance at 405 nm occurs due to BGL hydrolysis of 4-nitrophenyl-β-D-glucopyranoside (4-NPG) to 4-nitrophenol (4-NP). Clones selected for use as parents in genetic crosses are marked with black rectangles. Numerical data are listed in S1 Data.
