# Supplementary material for: Identification of genetic variants of the industrial yeast Komagataella phaffii (Pichia pastoris) that contribute to increased yields of secreted heterologous proteins
Source: PLoS Biol. 2022 Dec 15;20(12):e3001877. doi: 10.1371/journal.pbio.3001877 (PMC9754263; doi:10.1371/journal.pbio.3001877)
Supplement: S2 Fig — (A) Parental K. phaffii strains and diploids formed by mating. AMY429 and BY4742 are S. cerevisiae diploid and haploid strains, respectively, included as controls. Dashed red lines indicate haploid K. phaffii cell populations in the G1 and G2 phases of the cell cycle. (B, C) Ploidy screening of (B) superior segregants and (C) inferior segregants, from Cross 1. The top 2 histograms in each group show the DNA content of the diploid (CBS_BGL9/Pp2_BGL5, light red) and 1 haploid parent (Pp2_BGL5, blue) for comparison as diploid and haploid controls, respectively. Segregants that were not included in the final pools of 30 segregants are named in parentheses (segregants were excluded if they had diploid profiles, inconsistent rephenotyping results, or were simply supernumerary). (D, E) Ploidy screening of (D) superior segregants and (E) inferior segregants, from Cross 2. The top 2 histograms in each group show the DNA content of the diploid (CBS_BGL9/Pp4_BGL3, blue) and 1 haploid parent (Pp4_BGL3, red) for comparison as diploid and haploid controls, respectively. Segregants that were not included in the final pools of 30 segregants are named in parentheses. Raw data (FCS files) have been deposited in FlowRepository (https://flowrepository.org/). (PDF) [file pbio.3001877.s002.pdf]

A

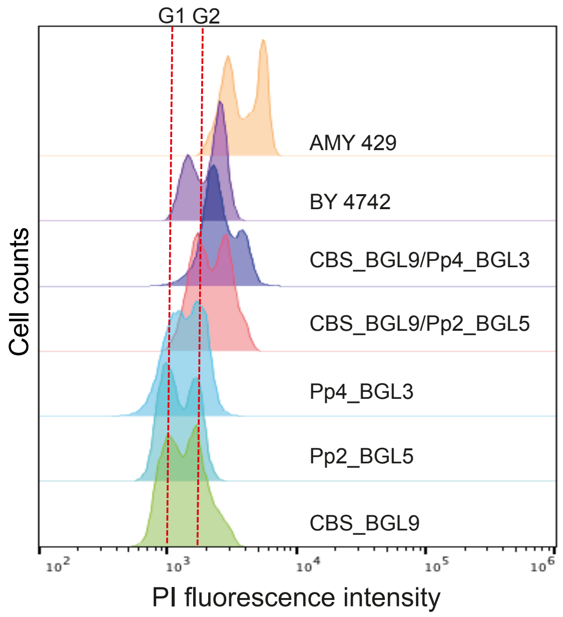

S2 Fig. Flow cytometric assessments of ploidy.
A, Parental *K. phaffii* strains and diploids formed by mating. AMY429 and BY4742 are *S. cerevisiae* diploid and haploid strains respectively, included as controls. Dashed red lines indicate haploid *K. phaffii* cell populations in the G1 and G2 phases of the cell cycle.
B, C, Ploidy screening of **B**, superior segregants and **C**, inferior segregants, from Cross 1. The top two histograms in each group show the DNA content of the diploid (CBS_BGL9/Pp2_BGL5, light red) and one haploid parent (Pp2_BGL5, blue) for comparison as diploid and haploid controls respectively. Segregants that were not included in the final pools of 30 segregants are named in parentheses (segregants were excluded if they had diploid profiles, inconsistent re-phenotyping results, or were simply supernumerary).
D, E, Ploidy screening of **D**, superior segregants, and **E**, inferior segregants, from Cross 2. The top two histograms in each group show the DNA content of the diploid (CBS_BGL9/Pp4_BGL3, blue) and one haploid parent (Pp4_BGL3, red) for comparison as diploid and haploid controls respectively. Segregants that were not included in the final pools of 30 segregants are named in parentheses.
Raw data (FCS files) have been deposited at FlowRepository (https://flowrepository.org/).

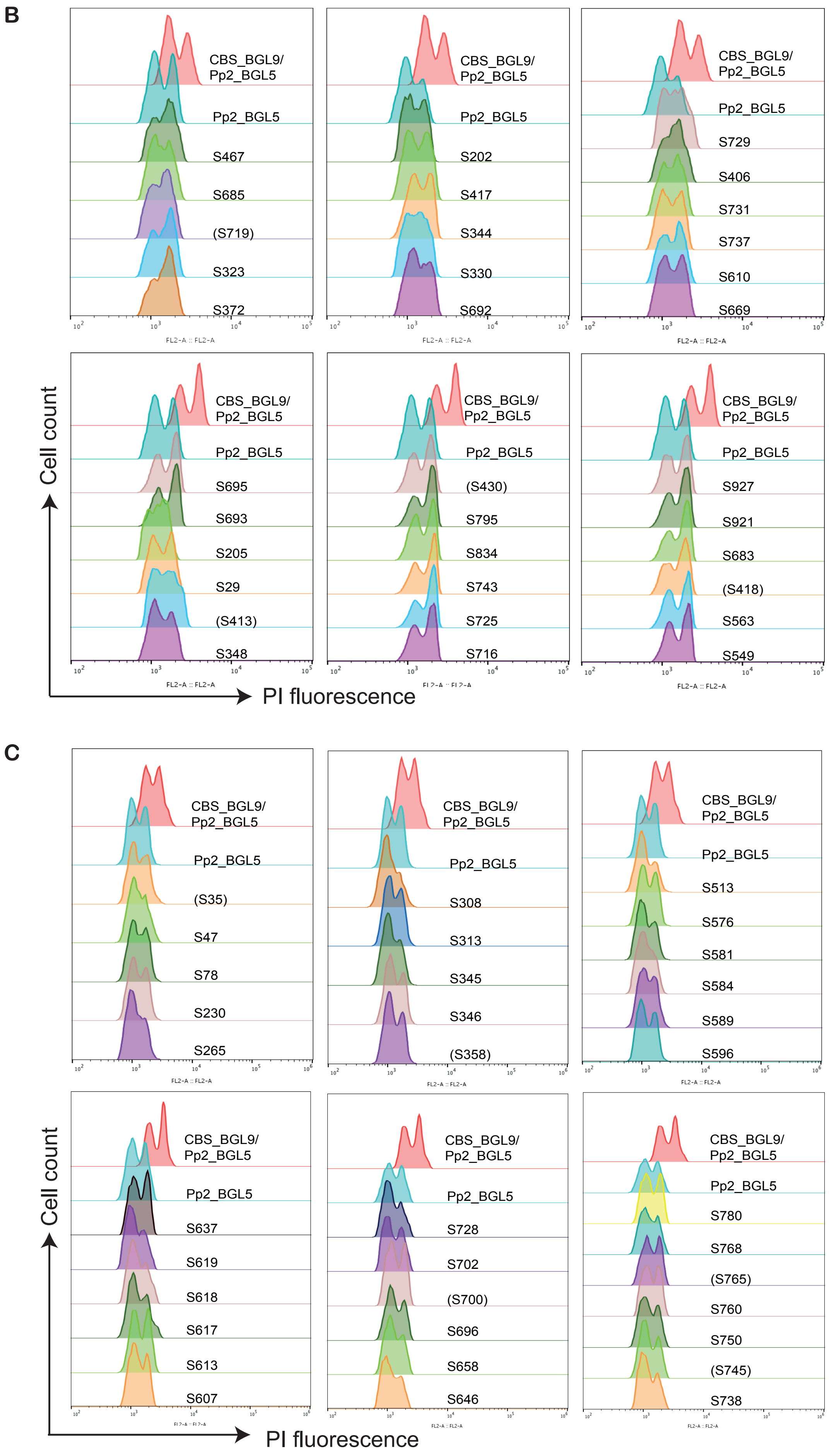

S2 Fig. (continued).
