# Supplementary material for: Identification of genetic variants of the industrial yeast Komagataella phaffii (Pichia pastoris) that contribute to increased yields of secreted heterologous proteins
Source: PLoS Biol. 2022 Dec 15;20(12):e3001877. doi: 10.1371/journal.pbio.3001877 (PMC9754263; doi:10.1371/journal.pbio.3001877)
Supplement: S3 Fig — (A, B) Distributions of BGL activity among all segregants in (A) Cross 1 and (B) Cross 2. The green lines show best-fit normal distribution curves. (C, D) Comparison of normalized 4-NP absorbance (BGL secretion) measurements in initial phenotyping and rephenotyping of segregants in (C) Cross 1 and (D) Cross 2. Red and green points denote inferior and superior segregants, respectively. (E, F) Comparison of normalized cell density (OD600) measurements in initial phenotyping and rephenotyping of segregants in (E) Cross 1 and (F) Cross 2. Red and green points denote inferior and superior segregants, respectively. Numerical data are listed in S1 Data. (PDF) [file pbio.3001877.s003.pdf]

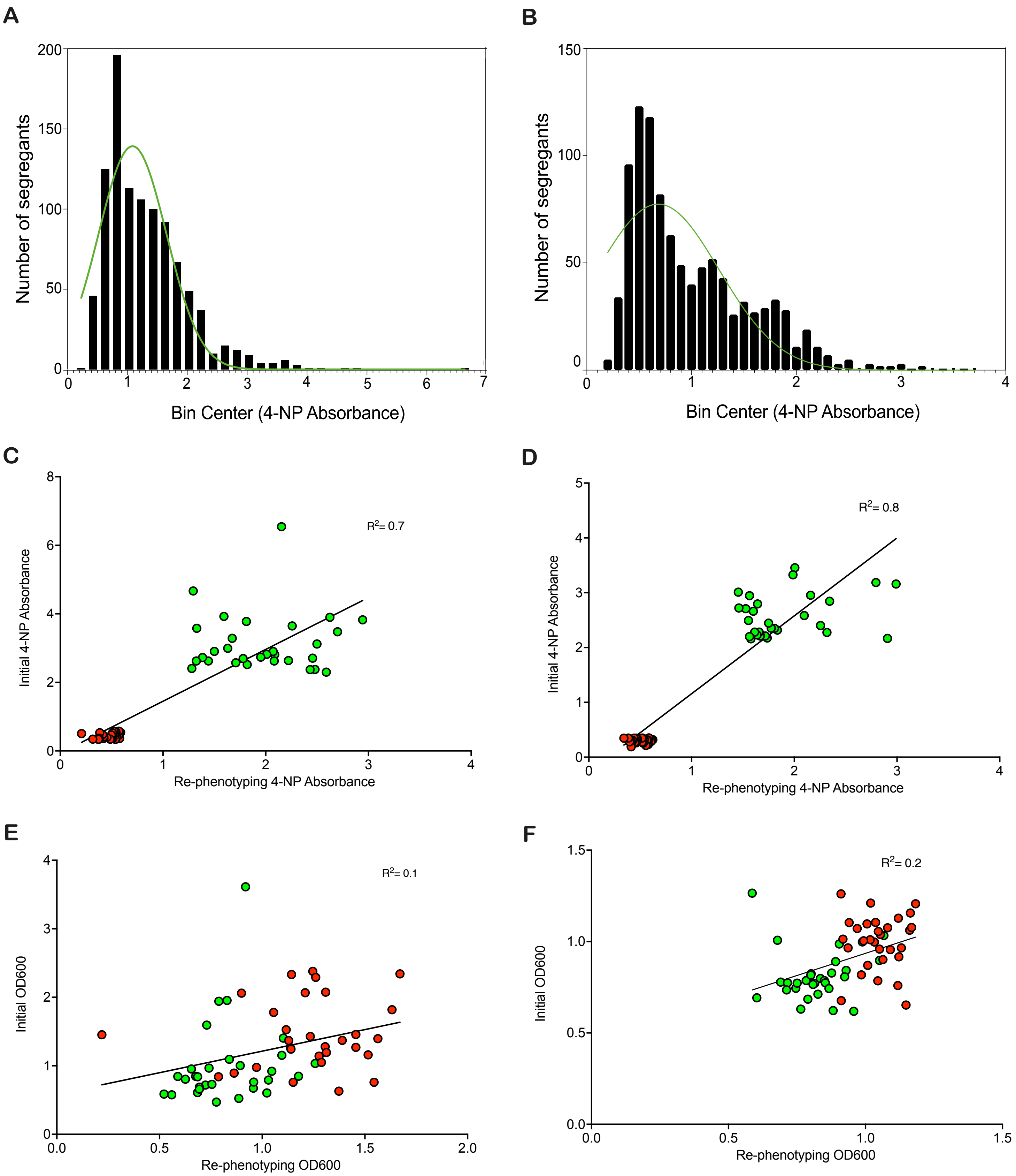

S3 Fig. Phenotypes of segregants.
A, B: Distributions of BGL activity among all segregants in **A**, Cross 1 and **B**, Cross 2. The green lines show best-fit normal distribution curves.
C, D: Comparison of normalized 4-NP absorbance (BGL secretion) measurements in initial phenotyping and re-phenotyping of segregants in **C**, Cross 1 and **D**, Cross 2. Red and green points denote inferior and superior segregants, respectively.
E, F: Comparison of normalized cell density (OD600) measurements in initial phenotyping and re-phenotyping of segregants in **E**, Cross 1 and **F**, Cross 2. Red and green points denote inferior and superior segregants, respectively. Numerical data are listed in S1 Data.
