# Supplementary material for: Identification of genetic variants of the industrial yeast Komagataella phaffii (Pichia pastoris) that contribute to increased yields of secreted heterologous proteins
Source: PLoS Biol. 2022 Dec 15;20(12):e3001877. doi: 10.1371/journal.pbio.3001877 (PMC9754263; doi:10.1371/journal.pbio.3001877)
Supplement: S4 Fig — (A) Pp2 allele frequencies in Cross 1. Dot plots show the location and estimated allele frequencies, obtained by bulk segregant sequencing, of Pp2 SNP alleles along the 4 chromosomes of K. phaffii. Green dots indicate the frequency of Pp2 SNP alleles in Illumina reads from the DNA pool of 30 superior segregants, whereas red dots indicate their frequency in the DNA pool of 30 inferior segregants. Broken magenta lines indicate the 50% allele frequency expected for loci unlinked to the BGL secretion phenotype. (B) Pp4 allele frequencies in Cross 2. Dot plots show the location and estimated allele frequencies, obtained by bulk segregant sequencing, of Pp4 SNP alleles along the 4 chromosomes of K. phaffii. Green dots indicate the frequency of Pp4 alleles in Illumina reads from the DNA pool of 30 superior segregants, whereas red dots indicate their frequency in the DNA pool of 30 inferior segregants. Numerical data are listed in S1 Data. (PDF) [file pbio.3001877.s004.pdf]

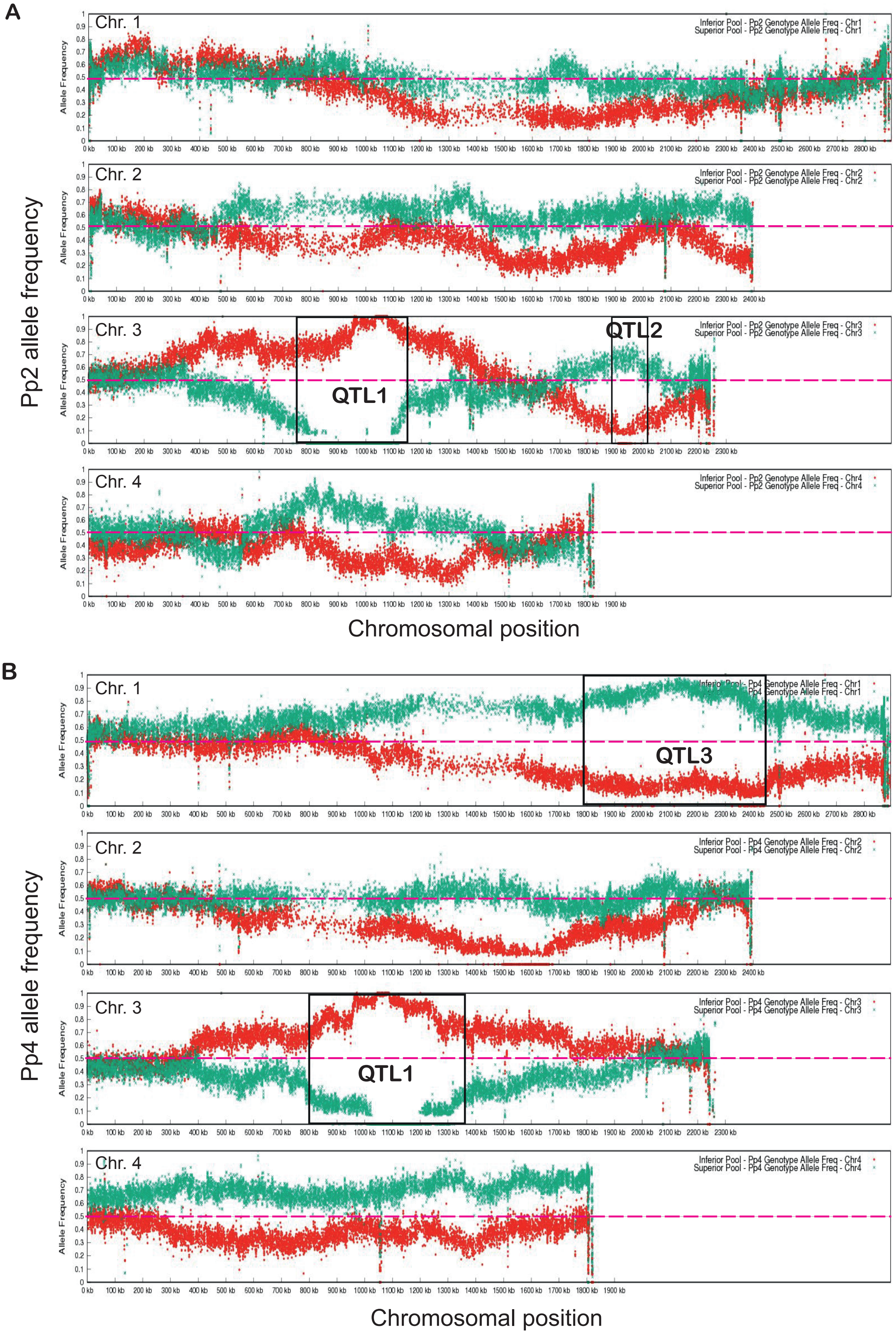

S4 Fig. Plots of SNP frequency versus chromosomal position in pooled (bulk) segregant analysis.
A, Pp2 allele frequencies in Cross 1. Dot plots show the location and estimated allele frequencies, obtained by bulk segregant sequencing, of Pp2 SNP alleles along the four chromosomes of K. phaffii. Green dots indicate the frequency of Pp2 SNP alleles in Illumina reads from the DNA pool of 30 superior segregants, whereas red dots indicate their frequency in the DNA pool of 30 inferior segregants. Broken magenta lines indicate the 50% allele frequency expected for loci unlinked to the BGL secretion phenotype.
B, Pp4 allele frequencies in Cross 2. Dot plots show the location and estimated allele frequencies, obtained by bulk segregant sequencing, of Pp4 SNP alleles along the four chromosomes of K. phaffii. Green dots indicate the frequency of Pp4 alleles in Illumina reads from the DNA pool of 30 superior segregants, whereas red dots indicate their frequency in the DNA pool of 30 inferior segregants. Numerical data are listed in S1 Data.
