# Supplementary material for: Identification of genetic variants of the industrial yeast Komagataella phaffii (Pichia pastoris) that contribute to increased yields of secreted heterologous proteins
Source: PLoS Biol. 2022 Dec 15;20(12):e3001877. doi: 10.1371/journal.pbio.3001877 (PMC9754263; doi:10.1371/journal.pbio.3001877)
Supplement: S5 Fig — Serial dilution spot test assays were used to compare the susceptibility to Calcofluor White (CFW, 20 μg/mL) of haploid K. phaffii strains with wild-type HOC1 and mutant hoc1 genes. (A) Unedited parental strains. (B) Edited strains. Genotypes at the HOC1 locus are indicated on the right. (PDF) [file pbio.3001877.s005.pdf]

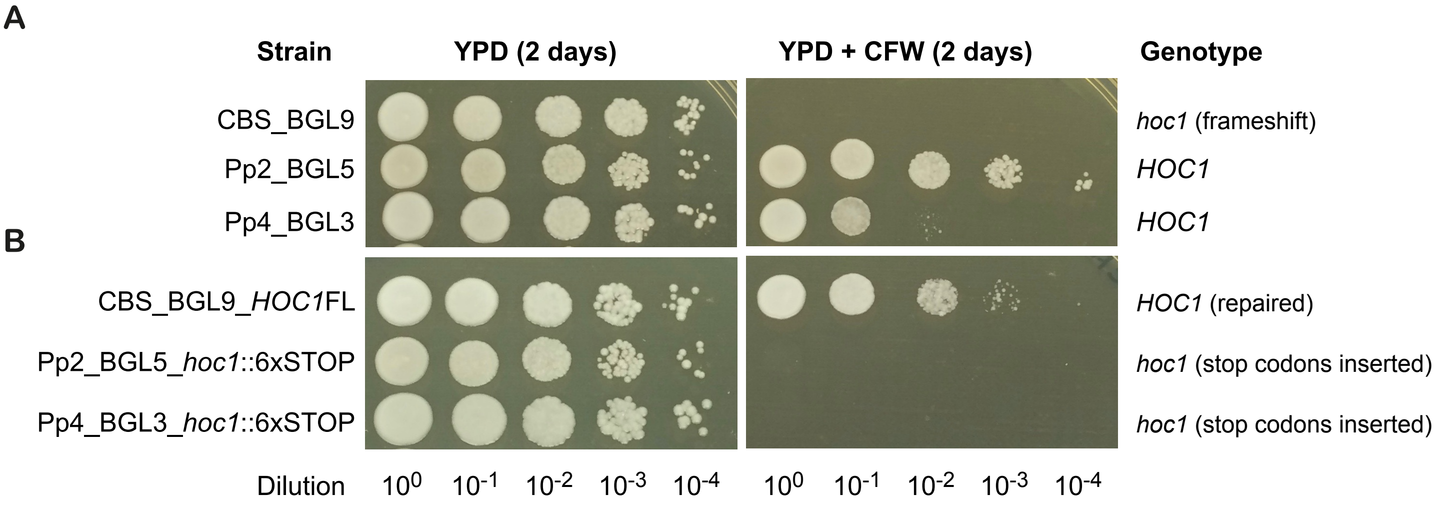


**S5 Fig.** *hoc1* mutants are sensitive to Calcofluor White. Serial dilution spot test assays were used to compare the susceptibility to Calcofluor White (CFW, 20 µg/mL) of haploid *K. phaffii* strains with wildtype *HOC1* and mutant *hoc1* genes. **A**, Unedited parental strains. **B**, Edited strains. Genotypes at the *HOC1* locus are indicated on the right.
