# Supplementary material for: Identification of genetic variants of the industrial yeast Komagataella phaffii (Pichia pastoris) that contribute to increased yields of secreted heterologous proteins
Source: PLoS Biol. 2022 Dec 15;20(12):e3001877. doi: 10.1371/journal.pbio.3001877 (PMC9754263; doi:10.1371/journal.pbio.3001877)
Supplement: S6 Fig — The data from the experiment in Fig 6 were reanalyzed to normalize BGL secretion (4-NP absorbance) by cell density (OD600) in each culture. Numerical data are listed in S1 Data. (PDF) [file pbio.3001877.s006.pdf]

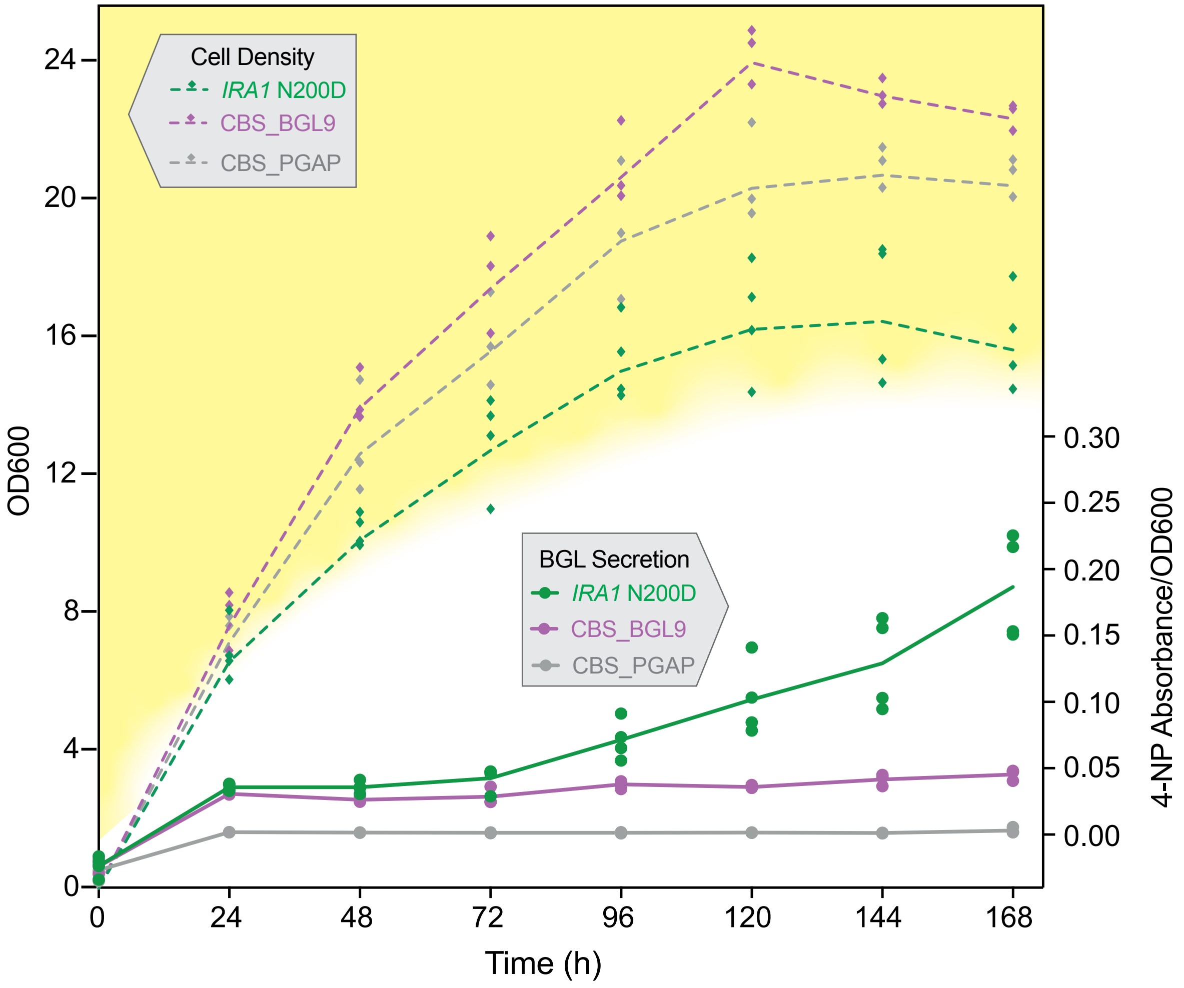

S6 Fig. Time course comparing OD600-normalised BGL secretion between CBS_BGL9 and its derivatives containing the IRA1N200D edit. The data from the experiment in Fig 6 was reanalyzed to normalize BGL secretion (4-NP absorbance) by cell density (OD600) in each culture. Numerical data are listed in S1 Data.
