# Supplementary material for: Identification of genetic variants of the industrial yeast Komagataella phaffii (Pichia pastoris) that contribute to increased yields of secreted heterologous proteins
Source: PLoS Biol. 2022 Dec 15;20(12):e3001877. doi: 10.1371/journal.pbio.3001877 (PMC9754263; doi:10.1371/journal.pbio.3001877)
Supplement: S7 Fig — Proteins were extracted from supernatants of 100-ml shake-flask cultures at 96 hours, 120 hours, 144 hours, and 168 hours. Comparisons are between CBS_PGAP (3 technical replicates), CBS_BGL9 (3 technical replicates), and ICs (4 independently clones in which the IRA1N200D variant was introduced by genome editing). The arrow indicates BGL, which migrates at its expected size of 120 kDa. L, molecular size standards. Due to a problem during gel loading, the sample from the fourth IC at 144 hours was split between 2 lanes. The cultures used for this experiment were the same ones used in Fig 6. (PDF) [file pbio.3001877.s007.pdf]

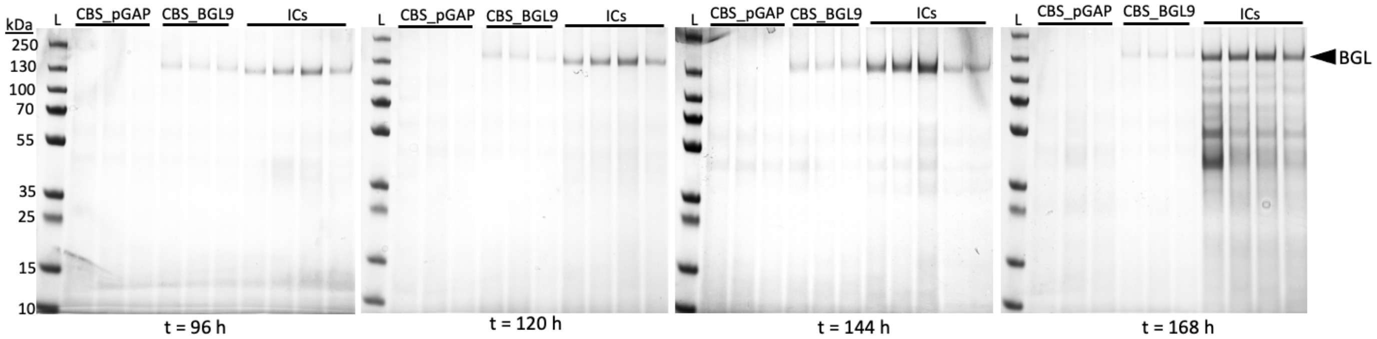

S7 Fig. SDS-PAGE analysis of BGL secretion over time in clones with and without *IRA1*^{N200D}. Proteins were extracted from supernatants of 100-ml shake flask cultures at 96 h, 120 h, 144 h and 168 h. Comparisons are between CBS_PGAP (3 technical replicates), CBS_BGL9 (3 technical replicates), and ICs (4 independently clones in which the *IRA1*^{N200D} variant was introduced by genome editing). The arrow indicates BGL, which migrates at its expected size of 120 kDa. L, molecular size standards. Due to a problem during gel loading, the sample from the fourth IC at 144 h was split between two lanes. The cultures used for this experiment were the same ones used in Fig 6.
