# Supplementary material for: Identification of genetic variants of the industrial yeast Komagataella phaffii (Pichia pastoris) that contribute to increased yields of secreted heterologous proteins
Source: PLoS Biol. 2022 Dec 15;20(12):e3001877. doi: 10.1371/journal.pbio.3001877 (PMC9754263; doi:10.1371/journal.pbio.3001877)
Supplement: S8 Fig — (A) Percentage viabilities indicate the proportion of cells that were determined by flow cytometry as exhibiting minimal intracellular accumulation of propidium iodide. Purple and gray points denote cell viability measurements in 3 independent cultures of CBS_BGL9 and CBS_pGAP, respectively. Green points denote 4 independently edited clones (ICs) in which the IRA1N200D substitution was introduced into the CBS_BGL9 background by genome editing. (B) Cell density values (OD600) determined at the same timepoints as in (A). These measurements were made on a set of 1-week 100 ml shake-flask cultures that replicated the conditions used in Fig 6. Numerical data are listed in S1 Data. (PDF) [file pbio.3001877.s008.pdf]

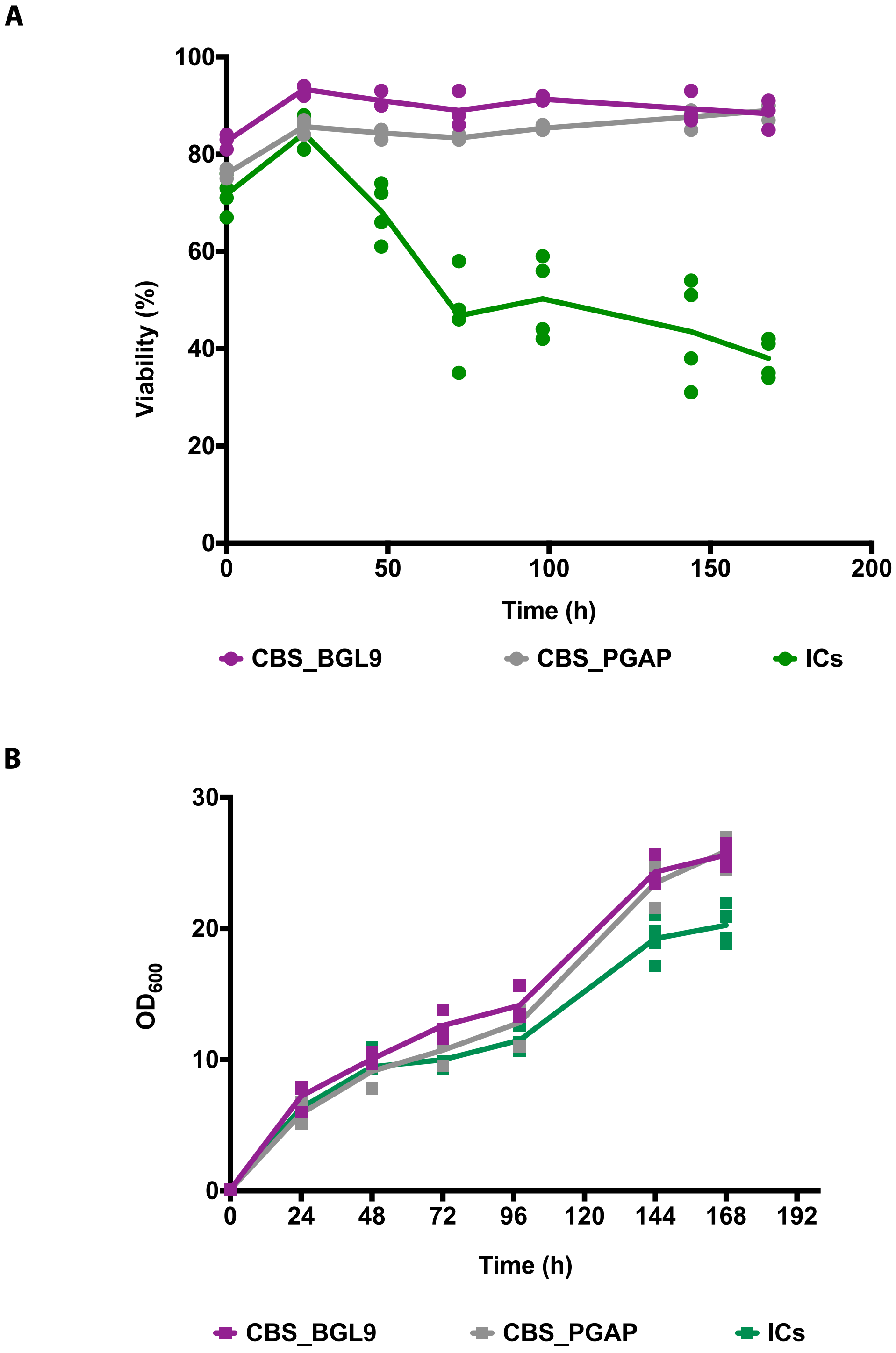

S8 Fig. Time-course assessment of cell viabilities during BGL secretion.

A, Percentage viabilities indicate the proportion of cells that were determined by flow cytometry as exhibiting minimal intracellular accumulation of propidium iodide. Purple and gray points denote cell viability measurements in three independent cultures of CBS_BGL9 and CBS_pGAP respectively. Green points denote four independently edited clones (ICs) in which the *IRA1*^{N200D} substitution was introduced into the CBS_BGL9 background by genome editing.

B, Cell density values (OD600) determined at the same timepoints as in (a). These measurements were made on a set of 1-week 100 ml shake flask cultures that replicated the conditions used in Fig 6.

Numerical data are listed in S1 Data.
