# Supplementary material for: Identification of genetic variants of the industrial yeast Komagataella phaffii (Pichia pastoris) that contribute to increased yields of secreted heterologous proteins
Source: PLoS Biol. 2022 Dec 15;20(12):e3001877. doi: 10.1371/journal.pbio.3001877 (PMC9754263; doi:10.1371/journal.pbio.3001877)
Supplement: S9 Fig — (A) β-galactosidase activity of clones of IT1005 incorporating the IRA1N200D edit (green dots, 7 independently edited clones) is compared to unedited clones from the same strain (orange squares, 2 independent clones) that were transformed for CRISPR editing but failed to incorporate the IRA1N200D SNP, and to the original unedited strain IT1005 (blue triangles, 7 technical replicates). The negative control strains are CBS_pGAP (open inverted triangles, 7 technical replicates) and its IRA1N200D derivative (brown diamonds, 3 technical replicates). (B) α-galactosidase activity of clones of IT1018 incorporating the IRA1N200D edit (green dots, 11 independently edited clones) is compared to unedited clones from the same strain (orange squares, 2 independent clones) that were transformed for CRISPR editing but failed to incorporate the IRA1N200D SNP, and to the original unedited strain IT1018 (blue triangles, 7 technical replicates). The negative controls are the same as in (A). Numerical data are listed in S1 Data. (PDF) [file pbio.3001877.s009.pdf]

A

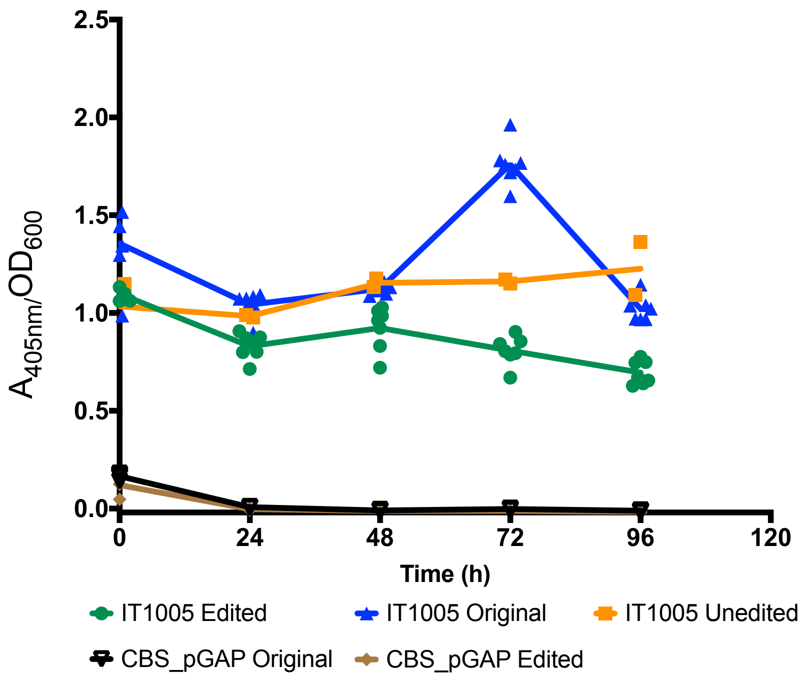

B

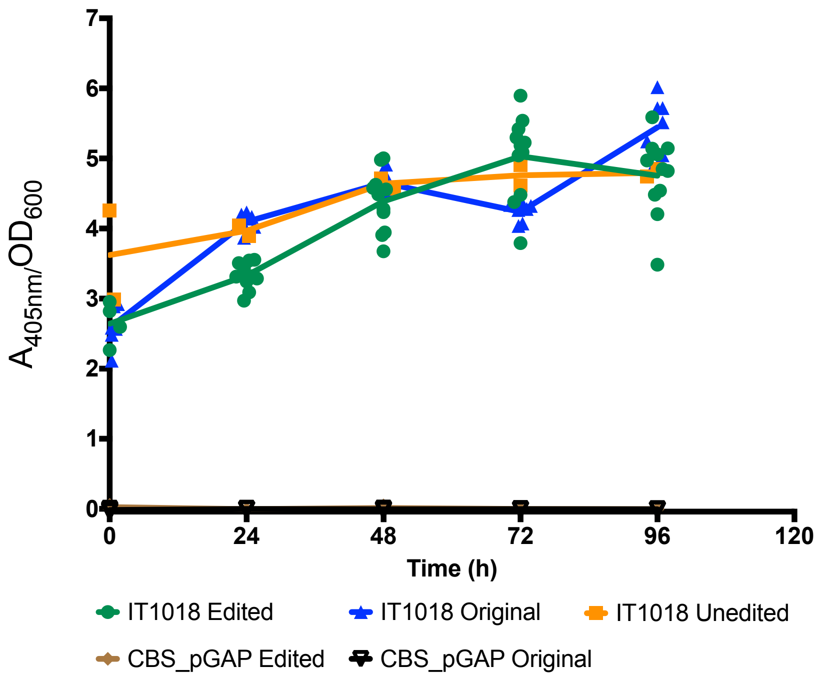

S9 Fig. Effect of the *IRA1*^{N200D} variant on secretion of galactosidase enzymes.
A, β-galactosidase activity of clones of IT1005 incorporating the *IRA1*^{N200D} edit (green dots, 7 independently edited clones) is compared to unedited clones from the same strain (orange squares, 2 independent clones) that were transformed for CRISPR editing but failed to incorporate the *IRA1*^{N200D} SNP, and to the original unedited strain IT1005 (blue triangles, 7 technical replicates). The negative control strains are CBS_pGAP (open inverted triangles, 7 technical replicates) and its *IRA1*^{N200D} derivative (brown diamonds, 3 technical replicates).
B, α-galactosidase activity of clones of IT1018 incorporating the *IRA1*^{N200D} edit (green dots, 11 independently edited clones) is compared to unedited clones from the same strain (orange squares, 2 independent clones) that were transformed for CRISPR editing but failed to incorporate the *IRA1*^{N200D} SNP, and to the original unedited strain IT1018 (blue triangles, 7 technical replicates). The negative controls are the same as in **A**.
Numerical data are listed in S1 Data.
