# Supplementary material for: Identification of genetic variants of the industrial yeast Komagataella phaffii (Pichia pastoris) that contribute to increased yields of secreted heterologous proteins
Source: PLoS Biol. 2022 Dec 15;20(12):e3001877. doi: 10.1371/journal.pbio.3001877 (PMC9754263; doi:10.1371/journal.pbio.3001877)
Supplement: S10 Fig — A section of a protein sequence alignment around position 200 of Ira1/2 proteins is shown. The arrow indicates the conserved asparagine (N) at position 200, which is substituted to aspartic acid (D) in K. phaffii isolate Pp2. This site corresponds to Asn226 of S. cerevisiae Ira1, and Asn253 of S. cerevisiae Ira2. The alignment was made using MUSCLE as implemented in Seaview v5.04 [59]. Colors indicate conservative amino acid groups. Sequence identifiers from MGOB [44] or NCBI are shown on the right. (PDF) [file pbio.3001877.s010.pdf]

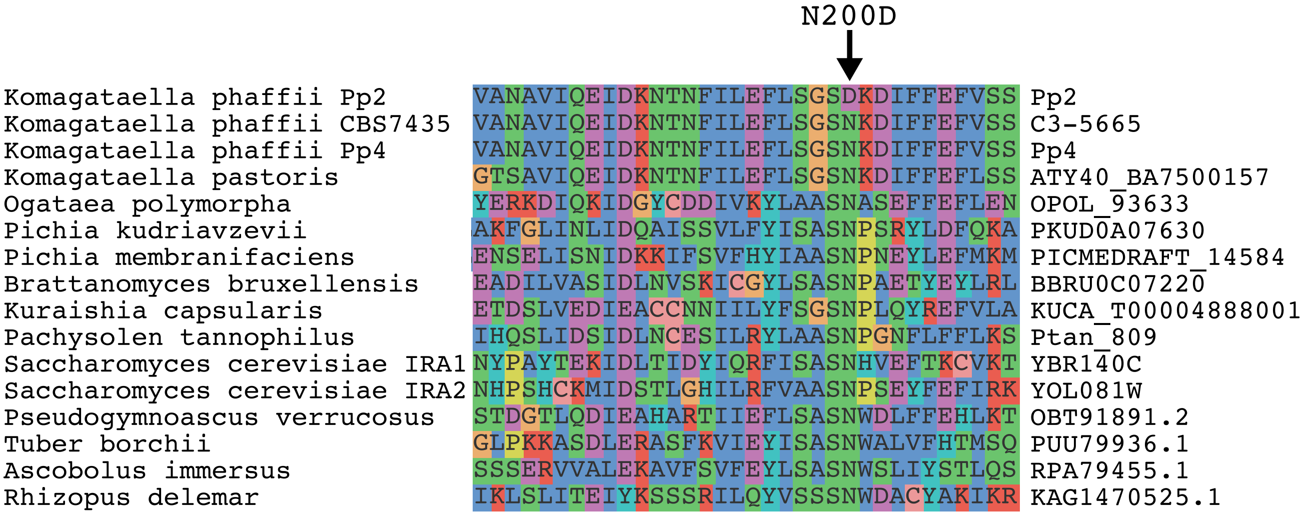

S10 Fig. Conservation of the Asn200 site in Ira1/2 proteins of fungi. A section of a protein sequence alignment around position 200 of Ira1/2 proteins is shown. The arrow indicates the conserved asparagine (N) at position 200, which is substituted to aspartic acid (D) in K. phaffii isolate Pp2. This site corresponds to Asn226 of S. cerevisiae Ira1, and Asn253 of S. cerevisiae Ira2. The alignment was made using MUSCLE as implemented in Seaview v5.04. Colors indicate conservative amino acid groups. Sequence identifiers from MGOB or NCBI are shown on the right.
