# Supplementary material for: Identification of genetic variants of the industrial yeast Komagataella phaffii (Pichia pastoris) that contribute to increased yields of secreted heterologous proteins
Source: PLoS Biol. 2022 Dec 15;20(12):e3001877. doi: 10.1371/journal.pbio.3001877 (PMC9754263; doi:10.1371/journal.pbio.3001877)
Supplement: S1 Table — (PDF) [file pbio.3001877.s011.pdf]

**S1 Table.** *Komagataella phaffii* strains used in this study.

| Strain | Description | Source |
|---|---|---|
| CBS7435<br>(NRRL Y-11430; CECT 11047) | Genome reference strain developed by Phillips Petroleum. Progenitor of strains GS115 and X-33. Inferred by Braun-Galleani et al. (2019) and Brady et al. (2020) to be derived from NRRL Y-7556 (CBS 2612), which is the type strain of *K. phaffii* and is a natural isolate from black oak (*Quercus kelloggii*), California, USA. Wild type. | Spanish Type Culture Collection (CECT) |
| Pp2 (NRRL Y-17741) | Natural isolate from Emory oak (*Quercus emoryi*), Arizona, USA. Wild type. | NRRL collection (USDA ARS) |
| Pp4 (NRRL YB-378) | Natural isolate from elm tree (*Ulmus americana*), USA. Wild type. | NRRL collection (USDA ARS) |
| CBS_PGAP | CBS7435 transformant harboring an integrated empty pGAPZα vector (control strain) | This study |
| CBS_BGL9 | CBS7435 transformant harboring BGL expression cassette | This study |
| Pp2_BGL5 | Pp2 transformant harboring BGL expression cassette | This study |
| Pp4_BGL3 | Pp4 transformant harboring BGL expression cassette | This study |
| CBS_BGL9/Pp2_BGL5 | Hybrid diploid isolated from CBS_BGL9 x Pp2_BGL5 cross | This study |
| CBS_BGL9/Pp4_BGL3 | Hybrid diploid isolated from CBS_BGL9 x Pp4_BGL3 cross | This study |
| CBS_BGL9_*HOC1FL* | CBS_BGL9 strain with corrected, full-length *HOC1* allele | This study |
| Pp2_BGL5_*HOC1DS* | Pp2_BGL5 strain with disrupted *HOC1* allele (6xSTOP tag) | This study |
| Pp4_BGL3_*HOC1DS* | Pp4_BGL3 strain with disrupted *HOC1* allele (6xSTOP tag) | This study |
| CBS_BGL9/Pp2_BGL5_*HOC1*DS | CBS_BGL9/Pp2_BGL5 diploid with the truncated (frameshifted) *HOC1* allele of CBS_BGL9 and disrupted *HOC1* allele of Pp2_BGL5 | This study |
| CBS_BGL9/Pp4_BGL3_*HOC1*DS | CBS_BGL9/Pp4_BGL3 diploid with the truncated (frameshifted) *HOC1* allele of CBS_BGL9 and disrupted *HOC1* allele of Pp4_BGL3 | This study |
| CBS_BGL9_*ira1*/Pp2_BGL5_*IRA1* | Hemizygote derivative of CBS_BGL9/Pp2_BGL5 harboring a disrupted *IRA1* allele of CBS_BGL9 | This study |
| CBS_BGL9_*IRA1*/Pp2_BGL5_*ira1* | Hemizygote derivative of CBS_BGL9/Pp2_BGL5 harboring a disrupted *IRA1* allele of Pp2_BGL5 | This study |
| CBS_BGL9_$IRA1^{N200D}$ | CBS_BGL9 strain harboring an *IRA1* N200D (c.598A>G) allele | This study |
| CBS_BGL9_$IRA1^{V393L}$ | CBS_BGL9 strain harboring an *IRA1* V393L (c.1177G>T) allele | This study |
| CBS_BGL9_$IRA1^{D399N}$ | CBS_BGL9 strain harboring an *IRA1* D399N (c.1195G>A) allele | This study |
| CBS_BGL9_$IRA1^{G1466D}$ | CBS_BGL9 strain harboring an *IRA1* G1466D (c.4397G>A) allele | This study |
| CBS_BGL9_$IRA1^{K404fs}$ | CBS_BGL9 strain harboring a frameshifted allele of *IRA1* at amino acid position 404 | This study |
| IT1005 | Strain expressing codon-optimized *Aspergillus niger* β-galactosidase from UPP constitutive promoter. BG08 genetic background (derivative of NRRL Y-11430). | Ilya Tolstorukov |
| IT1018 | Strain expressing codon-optimized *Aspergillus niger* α-galactosidase from UPP constitutive promoter. NRRL Y-11430 genetic background. | Ilya Tolstorukov |

NRRL, Northern Regional Research Laboratory, US Department of Agriculture; CBS, Centraalbureau voor Schimmelcultures (Westerdijk Institute), The Netherlands.
