# Supplementary material for: Identification of genetic variants of the industrial yeast Komagataella phaffii (Pichia pastoris) that contribute to increased yields of secreted heterologous proteins
Source: PLoS Biol. 2022 Dec 15;20(12):e3001877. doi: 10.1371/journal.pbio.3001877 (PMC9754263; doi:10.1371/journal.pbio.3001877)
Supplement: S2 Table — (PDF) [file pbio.3001877.s012.pdf]

S2 Table. Sequences of guide RNA targets, and primers used to amplify Repair Templates (RTs), for CRISPR/Cas9 editing of *HOC1* and *IRA1* genes.

(a) *HOC1* locus: Guide RNA targets and Repair Template primers for *HOC1* frameshift repair and *HOC1* disruption. In all primers, underlined nucleotides in red font indicate nucleotide substitutions introduced to alter PAM sites, and those in blue font indicate the 6x STOP codon cassette plus five additional nucleotides that together served as a reverse primer tag to screen for *HOC1* disruptants.

**Guide target/Primer**	**Sequence (5'→3')**	**Purpose**
**Guide RNA target 1**	**GATCATCCCGATGCTTTTTG**	Repairing *HOC1* frameshift.
Forward primer left piece of RT1	GAAGCTGATAATCGGCAGTACC	
Reverse primer left piece of RT1	CTGAAAATGTCTC<u>T</u>CCAAAAAGCATCGGGATGATCATTGGAATACAAAAC	
Forward primer right piece of RT1	GTTTTGTATTCCAATGATCATCCCGATGCTTTTTGG<u>A</u>GAGACATTTTCAG	
Reverse primer right piece of RT1	CAGGTTAGATTTCTAAGGAACC	
**Guide RNA target 2**	**AACAACTCATGAACGAAACC**	Disruption of *HOC1*.
Forward primer left piece of RT2	AATGTCAGCTAGTTCCAGTGAG	
Reverse primer left piece of RT2	GGCTTCGAAGAAT<u>G</u>CTGGT<u>TAATGATGATAGTAATAGCGAGC</u>TTCGTTCATGAGTTG	
Forward primer right piece of RT2	CAACTCATGAACGAA<u>GCTCGCTATTACTATCATCATTA</u>ACCAG<u>C</u>ATTCTTCGAAGCC	
Reverse primer right piece of RT2	CAGGTTAGATTTCTAAGGAACC	

(b) *IRA1* locus: Guide RNA targets and Repair Template primers for SNP editing in *IRA1.* In all primers, underlined nucleotides in red font indicate nucleotide substitutions introduced to alter PAM sites, and those in blue font indicate incorporated SNPs.

**Guide target/Primer**	**Sequence (5′→3′)**	**Purpose**
**Guide RNA target 1**	**ACAAGACAAAATGGAACCTG**	Editing the 598A>G SNP (N200D). To introduce this SNP, the Right piece of RT1 was amplified from Pp2 genomic DNA.
Forward primer left piece of RT1	ATGAGTGAATCTTTAGTGGCATC	
Reverse primer left piece of RT1	CTCTTGAATAACTGCATTGGCTACAGCAGGTTCCATTTTGTCTTGTTTAAAC	
Forward primer right piece of RT1	GTTTAAACAAGACAAAATGGAACCTGCTGTAGCCAATGCAGTTATTCAAGAG	
Reverse primer right piece of RT1	CTTTGAGGCAAGCTTCACCAAGGCATTGTCAGGCCTATAAACAGACAATATGGC	
**Guide RNA target 2**	**TGACAATGCCTTGGTGAAGC**	Editing the 1177G>T SNP (V393L).
Forward primer left piece of RT2	CTTTGAGTTCGTATCTAGCAAG	
Reverse primer left piece of RT2	CTTTGAGGCAAGCTTCACCAAGGCATTGTCAGGCCTATAAACAGACAATATGGC	
Forward primer right piece of RT2	GCCATATTGTCTGTTTATAGGCCTGACAATGCCTTGGTGAAGCTTGCCTCAAAG	
Reverse primer right piece of RT2	GTAACATATCAACCAATGAATCG	
Forward primer left piece of RT3	CTTTGAGTTCGTATCTAGCAAG	Editing the 1195G>A SNP (D399N).
Reverse primer left piece of RT3	CTTTGAGGCAAGCTTCACCAAGGCATTGTTAGGCCTATAAACAGACACTATGGC	
Forward primer right piece of RT3	GCCATAGTGTCTGTTTATAGGCCTAACAATGCCTTGGTGAAGCTTGCCTCAAAG	
Reverse primer right piece of RT3	GTAACATATCAACCAATGAATCG	
**Guide RNA target 3**	**TACATATGACAACGAACTGT**	Editing the 4397G>A SNP (G1466D). To introduce this SNP, the Left piece of RT4 was amplified from Pp2 genomic DNA.
Forward primer left piece of RT4	ACAGCTACTAATTGAATATGGC	
Reverse primer left piece of RT4	GCTTTTGTGGGTGGCTAACAGTTCGTTGTCATATGTAGAGACAACATCGATA	
Forward primer right piece of RT4	TATCGATGTTGTCTCTACATATGACAACGAACTGTTAGCCACCCACAAAAGC	
Reverse primer right piece of RT4	GGAGGAATTAGTGAGTTGAGAC	
