# Supplementary figures and images for: Identification of genetic variants of the industrial yeast Komagataella phaffii (Pichia pastoris) that contribute to increased yields of secreted heterologous proteins
Source: PLoS Biol. 2022 Dec 15;20(12):e3001877. doi: 10.1371/journal.pbio.3001877 (PMC9754263; doi:10.1371/journal.pbio.3001877)

Original gel images for S7 Fig.

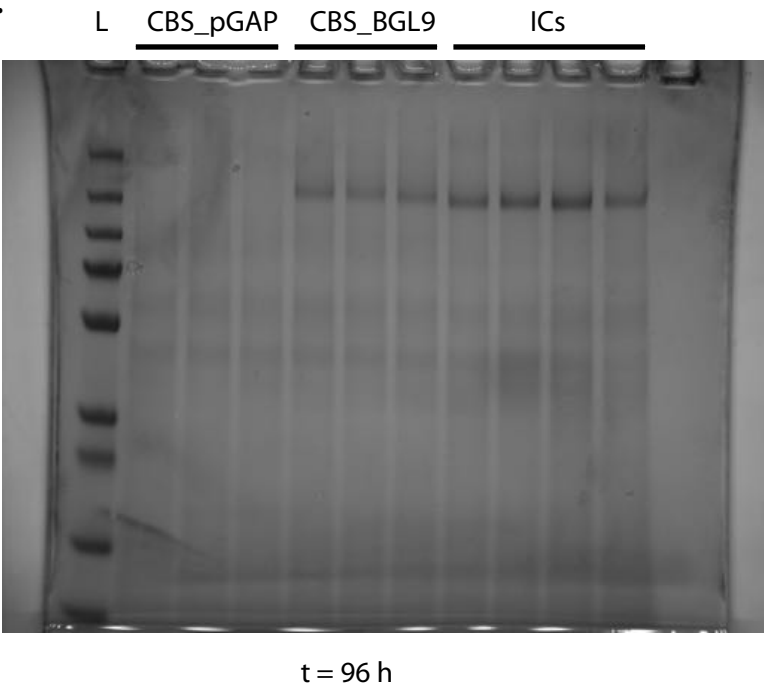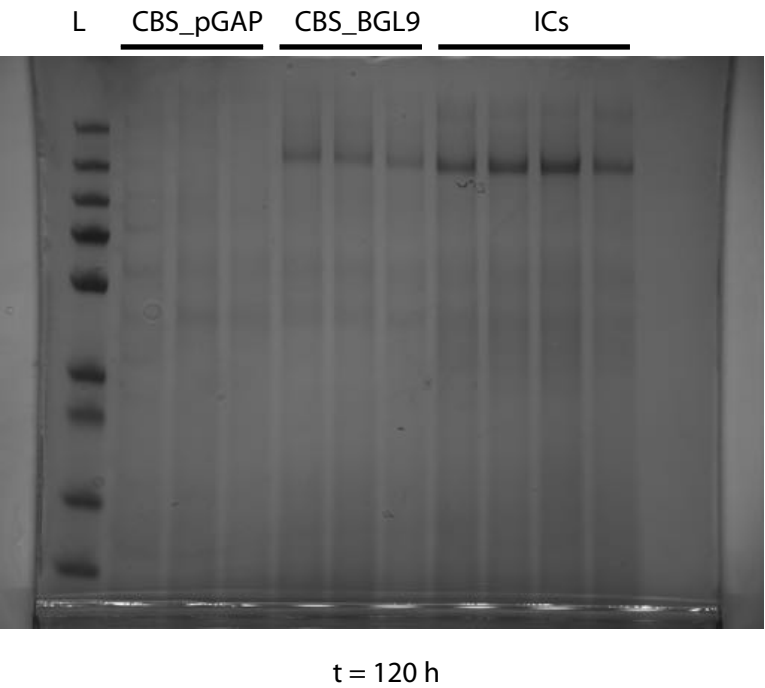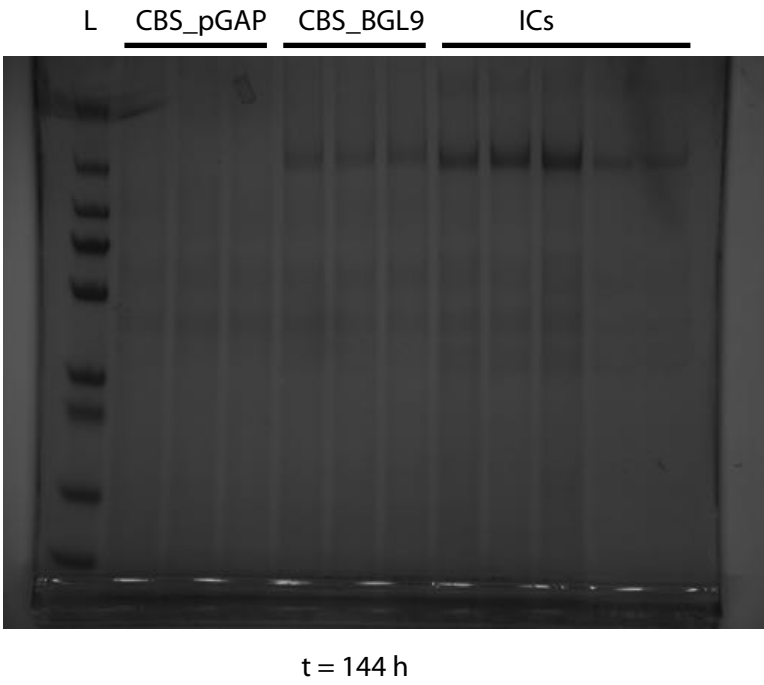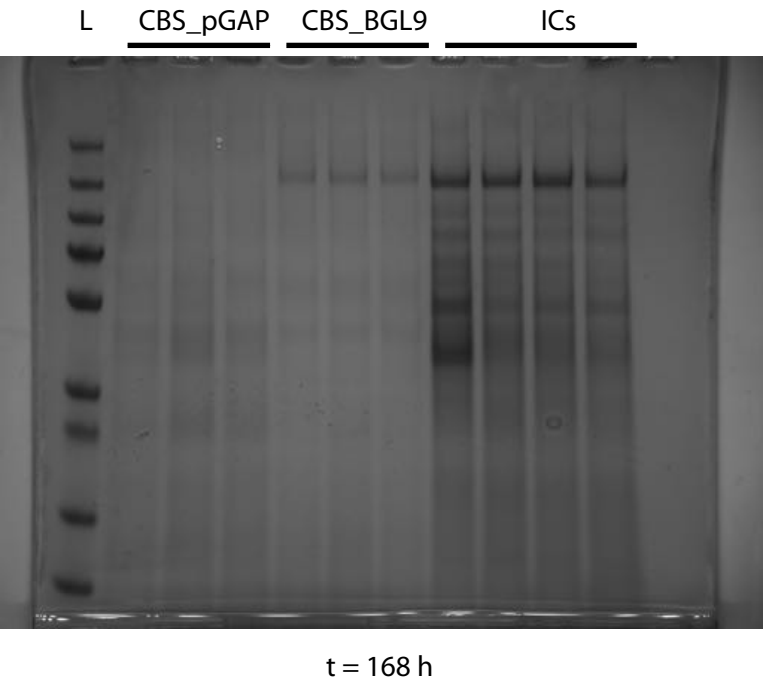

Supplement: S1 Raw Images — (PDF) [file pbio.3001877.s017.pdf]
